# Environmental particulate reference materials increase DCFDA fluorescence and reduce ATP-dependent viability in primary mouse retinal cells

**DOI:** 10.1016/j.bbrep.2026.102758

**Published:** 2026-08-26

**Authors:** Wanjing Chen, Yoko Iizuka, Fumihiko Mabuchi, Satoko Yoshizawa, Tomoko Muramatsu, Kenji Kashiwagi

**Affiliations:** Department of Ophthalmology, Faculty of Medicine, University of Yamanashi, 1110 Shimokato, Chuo, Yamanashi, 409-3898, Japan

**Keywords:** Particulate matter, Retinal ganglion cell, Retinal glial cell, Reactive oxygen species

## Abstract

Long-term exposure to ambient particulate matter ≤2.5 μm in aerodynamic diameter (PM2.5) has been associated with optic neuropathy and retinal neurodegeneration, but experimental evidence remains limited. We assessed two certified environmental particulate reference materials in primary mouse retinal cells and after repeated ocular-surface exposure in mice. For the in vitro analyses, technical replicate wells were first averaged within each independent cell isolation, and each experimental condition was then normalized to the corresponding control from the same biological replicate. Particulate exposure produced condition-dependent changes in 2′,7′-dichlorofluorescin diacetate (DCFDA) fluorescence and ATP-dependent viability, with statistically significant effects concentrated in selected retinal glial-cell conditions. After within-experiment normalization and multiplicity adjustment, direct comparisons did not demonstrate a statistically significant protective effect of α-tocopherol or N-acetylcysteine on particulate-associated responses, and retinal glial-cell coculture did not significantly improve RGC survival relative to monoculture. In postnatal mice, topical particulate administration was associated with inner retinal thinning and lower RGC-marker-positive cell counts. In adult Thy1-CFP mice, neither sample-specific nor post hoc pooled within-mouse comparisons with contralateral saline-treated eyes were statistically significant. A separate post hoc exploratory between-animal comparison showed fewer CFP-positive cells in pooled particulate-treated eyes than in independent bilateral-saline controls (mean difference, −14.4 cells; 95% CI, −28.1 to −0.7; P = 0.040). These findings support cellular and retinal susceptibility under the conditions used, but they do not establish particle transport to the retina, a causal oxidative mechanism, or quantitative risk from ambient PM2.5 exposure in humans.

## Highlights

• Particulates altered DCFDA fluorescence and ATP-dependent viability in retinal cells.
• Antioxidants did not show statistically significant rescue after adjusted analyses.
• Glial coculture did not significantly improve RGC survival after particulate exposure.
• Topical particulate exposure was associated with inner retinal changes in mice.

## Graphical abstract

**Figure:**
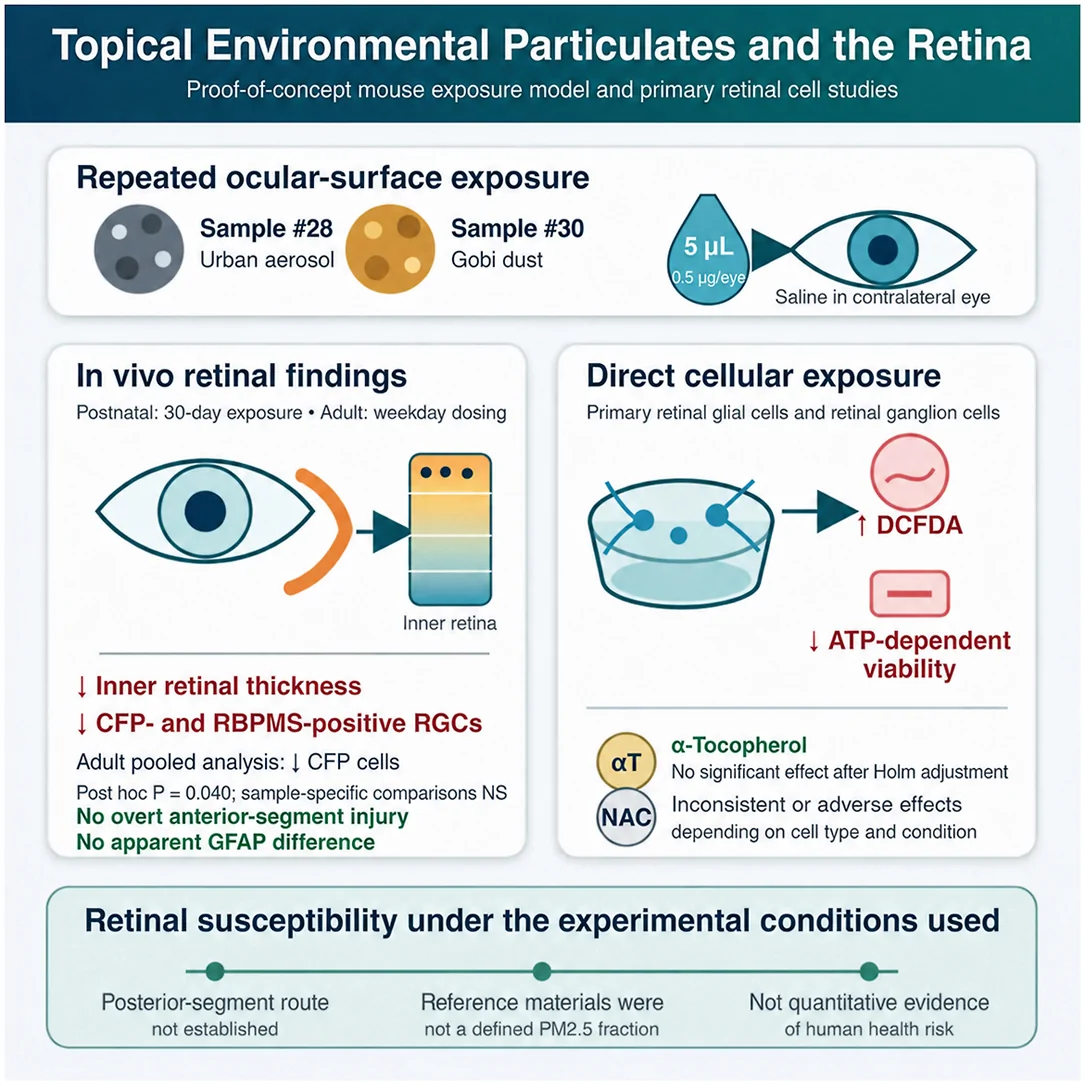

## 1 Introduction

Air pollution-related health risks have emerged as a significant global concern [1]. Among various air pollutants, atmospheric particulate matter (PM) has been identified as one of the most robust and consistent predictors of mortality. An expanding body of research has linked exposure to ambient PM with a range of systemic disorders, including cardiovascular disease [2,3], respiratory dysfunction [4], neurological disorders [5], and malignancies [[6], [7], [8]].

The eye is a uniquely exposed sensory organ and is therefore susceptible to airborne environmental pollutants. Previous studies have shown that air pollution is associated with ocular-surface disorders, including dry eye [9,10], conjunctivitis [11], and corneal inflammation [12,13]. In addition to anterior segment involvement, there is growing concern that air pollutants may affect deeper ocular structures, including the retina and optic nerve. Epidemiological studies have also reported associations between air pollution and optic neuropathies such as glaucoma [[14], [15], [16], [17], [18], [19], [20], [21], [22], [23], [24]].

Experimental studies have begun to identify retinal effects of PM2.5. Gu et al. reported that PM2.5 impaired the inner blood–retinal barrier through inflammatory and ferroptotic responses in retinal vascular endothelial cells, whereas Li et al. demonstrated visual dysfunction and retinal neuronal degeneration after systemic airborne exposure [18,20]. Nevertheless, the retinal consequences of direct ocular-surface contact and the relative responses of RGCs and retinal glial cells remain incompletely characterized.

PM2.5 is defined as particulate matter with an aerodynamic diameter of 2.5 μm or less. Because of its small size, inhaled PM2.5 can penetrate deeply into the respiratory tract and may enter the circulation [25]. PM2.5 is a complex mixture of organic compounds, metals, and other constituents that can promote oxidative stress and inflammatory or cell-death pathways [13,26], as also reported in human corneal epithelial cells exposed to particulate matter [27,28]. Such pathways may be relevant to retinal ganglion cell (RGC) degeneration and optic nerve injury, although the route and relevance of direct ocular-surface exposure remain uncertain.

In this study, we examined whether repeated ocular-surface exposure to two certified environmental particulate reference materials was associated with structural changes in the mouse inner retina. Complementary in vitro experiments evaluated responses of isolated primary RGCs and retinal glial cells, including reactive oxygen species (ROS), ATP-dependent viability, antioxidant modulation, and RGC survival in an RGC–glial coculture system. This design was intended to characterize retinal susceptibility and cell type-specific responses; it was not designed to reproduce ambient exposure levels or establish the route of particle transport from the ocular surface to the retina.

## 2 Methods

### 2.1 Animals

Two types of mice were used: wild-type C57BL/6J mice and Cg-Tg(Thy1-CFP)23Jrs/J mice (Thy1-CFP mice), in which RGCs express cyan fluorescent protein (CFP). All procedures were conducted in accordance with the ARVO Statement for the Use of Animals in Ophthalmic and Vision Research and were approved by the Institutional Animal Care and Use Committee of the University of Yamanashi (approval number A3-26). Efforts were made to minimize the number of animals used in the experiments and animal suffering.

The numbers of animals initially used in each experiment are described in the relevant Methods subsections. Samples that could not be evaluated reliably because of tissue-processing problems, inadequate section or image quality, or other documented technical issues were excluded from the corresponding analysis. Exclusions were based on technical evaluability rather than on the magnitude or direction of the observed results. Consequently, the final number of animals included in the analysis varied among experiments and outcome measures; the analyzed n is reported in the Results and figure legends.

### 2.2 Particulate reference materials and exposure rationale

Two certified environmental reference materials obtained from the National Institute for Environmental Studies (NIES) were selected: NIES CRM #28 Urban Aerosols (hereafter #28), collected from ventilation filters in central Beijing, and NIES CRM #30 Gobi Kosa Dust (hereafter #30), prepared from mineral dust collected in the Gobi Desert. CRM #28 was sieved through a 32-μm mesh, and 99% of particles by number were reported to be < 10 μm. CRM #30 was prepared as a <10-μm fraction and has an estimated median aerodynamic diameter of approximately 4 μm [29,30]. Neither material represents an exclusively ≤2.5-μm fraction. Their major elemental compositions are shown in Table 1. The materials were selected to compare particles with different sources and compositions rather than to identify a single compositional determinant of toxicity. The eye-drop model was designed as a proof-of-concept model of direct ocular-surface contact and not as a quantitative simulation of airborne or inhalational exposure. A suspension concentration of 100 μg/mL (5 μL; 0.5 μg/eye per administration) was selected to permit reproducible topical dosing. The administered dose cannot be directly converted to an ambient atmospheric concentration or cumulative human exposure.

**Table 1:** Elemental composition of the particulate reference materials.

Element	#30 Gobi Kosa Dust	#28 Urban Aerosols
Aluminum (%)	7.58	5.04
Calcium (%)	4.25	6.69
Iron (%)	3.84	2.92
Magnesium (%)	1.51	1.4
Potassium (%)	2.13	1.37
Sodium (%)	0.939	0.796
Titanium (%)	0.426	0.292
Barium (mg/kg)	535	874
Manganese (mg/kg)	768	686
Strontium (mg/kg)	250	469
Zinc (mg/kg)	93.1	1100
Lead (mg/kg)	22.4	403
Copper (mg/kg)	34.1	104
Nickel (mg/kg)	29.1	63.8
Uranium (mg/kg)	2.62	4.33

### 2.3 Topical particulate exposure in postnatal mice

The eye-drop experiment modeled direct ocular-surface contact with particulate material rather than ambient airborne exposure. A 5-μL volume of particulate suspension (100 μg/mL in saline; 0.5 μg/eye per day) was applied once daily to the experimental eye, and the contralateral control eye received the same volume of saline. The suspensions were vortexed immediately before administration to minimize particle sedimentation. A total of 52 wild-type mice were used in the in vivo experiments. Of these, 25 mice underwent the 30-day RBPMS experiment described below, while the remaining mice were used in the short-term histological and trace-element experiments. Mice used for short-term histological assessment were treated for 3–10 days beginning between postnatal days 5 and 15 and were euthanized at the end of each experiment for H&E evaluation.

For the longer-term structural assessment, 12 postnatal Thy1-CFP mice, comprising approximately equal numbers of males and females, were allocated to the sample #28 group (n = 6) or the sample #30 group (n = 6). One eye of each mouse received the particulate suspension once daily for 30 days beginning on postnatal day 6, while the contralateral eye received saline. Three mice in the sample #30 group were excluded from RGC quantification because tissue-processing problems precluded reliable analysis. Consequently, the final analysis included six mice in the sample #28 group and three mice in the sample #30 group. These postnatal mice were distinct from the 15 adult Thy1-CFP mice used in the adult exposure experiment. For the 30-day RBPMS analysis, 25 postnatal wild-type mice were allocated to the sample #28 group (n = 11) or the sample #30 group (n = 14). Beginning on postnatal day 6, one eye of each mouse received the particulate suspension once daily for 30 days, while the contralateral eye received saline. These 25 mice were included in the total of 52 wild-type mice used in the in vivo experiments. Because both eyes belonged to the same animal, inadvertent contralateral exposure through grooming or systemic redistribution cannot be excluded.

### 2.4 Preparation of paraffin sections and hematoxylin and eosin (H&E) staining

Wild-type mouse eyes were enucleated and fixed overnight in 4% paraformaldehyde in phosphate-buffered saline (PBS) at 4°C. After dehydration through a graded ethanol series and clearing, the eyes were embedded in paraffin (Tissue-Tek® Paraffin Wax II 60; Sakura Finetek Japan Co., Ltd., Tokyo, Japan), and 4-μm-thick sections were prepared. The sections were deparaffinized in Hemo-De (CS-1001-4; FALMA, Tokyo, Japan), rehydrated through a graded ethanol series, and stained with hematoxylin (30011; MUTO PURE CHEMICALS Co., Ltd., Tokyo, Japan) and eosin (058-00062; FUJIFILM Wako Pure Chemical Corporation, Osaka, Japan). The sections were then dehydrated, cleared, and mounted with coverslips. All quantitative evaluations were performed by investigators masked to the experimental conditions.

### 2.5 Retinal thickness measurement and cell counting in the ganglion cell layer

Retinal images were captured using a BZ-X700 microscope (KEYENCE, Osaka, Japan). Four non-overlapping sections were selected from the central region between the optic disc and the retinal periphery in each eye. Retinal thickness was measured using ImageJ software (version 1.53t; National Institutes of Health, Bethesda, MD, USA), and the mean value for each eye was calculated.

Cells in the ganglion cell layer (GCL) within the same central retinal region were counted on H&E-stained sections. For each section, a rectangular region of 650 × 250 pixels (approximately 1.67 mm × 0.64 mm) was selected for manual counting. Regions of interest were defined at a fixed distance from the optic nerve head (500–800 μm), and the same sampling strategy was applied to all eyes. Four non-overlapping areas were analyzed per eye, and the mean GCL cell count per area was calculated. Because the GCL also contains displaced amacrine cells, these H&E-based counts were not considered RGC-specific; RGC-specific conclusions were based on CFP and RNA binding protein with multiple splicing (RBPMS) measurements.

To assess ocular tissue toxicity following topical administration of the particulate suspensions, the cornea, sclera, iris, ciliary body, lens, and retina were examined for overt structural abnormalities and inflammatory cell infiltration on routine histological examination. Representative ocular histology is shown in Supplemental Fig. 1.

### 2.6 Cryosection preparation and RGC quantification in postnatal mice

After 30 days of topical particulate administration, the eyes were enucleated and fixed overnight in 4% paraformaldehyde in PBS at 4°C. After fixation, the anterior segment and lens were removed, and the eyecups were cryoprotected by sequential immersion in 10%, 20%, and 30% sucrose solutions (w/v in PBS) at 4°C. The tissues were embedded in Tissue-Tek® O.C.T. compound (Sakura Finetek Japan Co., Ltd., Tokyo, Japan) and sectioned at a thickness of 4 μm using a cryostat (Leica CM1520; Leica Biosystems, Tokyo, Japan). RGCs were evaluated using two complementary markers. CFP-positive cells were identified directly in retinal cryosections from Thy1-CFP mice, and RNA-binding protein with multiple splicing (RBPMS)-positive RGCs were identified by immunofluorescence staining with an anti-RBPMS antibody (ab194213; Abcam, Cambridge, UK). Nuclei were counterstained with DAPI (sc-24941; Santa Cruz Biotechnology, Dallas, TX, USA). CFP-positive and RBPMS-positive cells were counted at three predefined sampling locations in the central retina, and the counts from the three locations were summed to yield one value per eye. Fluorescence images were acquired using a BZ-X700 fluorescence microscope (KEYENCE), and cells were manually quantified using ImageJ. Image acquisition and cell counting were performed with the investigators masked to the experimental conditions.

### 2.7 Topical particulate exposure in adult Thy1-CFP mice

A total of 15 Thy1-CFP mice (7 males and 8 females; 11–20 weeks of age) were allocated to the following three groups (n = 5 per group): bilateral saline treatment, sample #28 administered to one eye with saline administered to the contralateral eye, or sample #30 administered to one eye with saline administered to the contralateral eye. Allocation was stratified to achieve an approximately balanced distribution of sex and age across the three groups.

A 1 mg/mL stock suspension was diluted tenfold with sterile saline to a final concentration of 0.1 mg/mL (100 μg/mL). Five microliters of particulate suspension (0.5 μg/eye per administration) or saline was instilled on each dosing day. Separate pipettes were used for the particulate and saline preparations to minimize cross-contamination.

A 5-μL volume of the test suspension was topically administered once daily on weekdays over a 15-day period, for a total of 11 administrations. On the day after the final administration, the mice were euthanized. The superior aspect of each eye was marked before enucleation to maintain tissue orientation. The eyes were washed with PBS and fixed overnight in 500 μL of 4% paraformaldehyde in PBS at 4°C. After removal of the anterior segment and lens, the eyecups were washed with PBS and incubated sequentially in 10%, 20%, and 30% sucrose in PBS for 1 h each. The tissues were immersed in O.C.T. compound for 5 min, embedded with the superior aspect oriented downward, frozen on dry ice, and stored at −80°C. Cryosections were prepared from the superior side of the eye. Four 4-μm-thick tissue sections were mounted on each slide. Five slides were selected from each eye at intervals of five slides to minimize double counting of the same cells. As in postnatal mice, CFP-positive cells were counted at three predefined sampling locations in the central retina, and the counts from all three locations were summed for each section. Fluorescence images were acquired at ×20 magnification using a BZ-X700 fluorescence microscope (KEYENCE), and cells were manually quantified using ImageJ. Image acquisition and cell counting were performed by an investigator masked to treatment allocation. CFP-positive cell counts were used as an indicator of RGC abundance rather than as a direct measure of cell viability. Measurements from all analyzed sections were aggregated to yield one value per eye, and each mouse constituted one experimental unit. For the bilateral-saline group, the two eyes of each mouse were averaged, yielding five independent animal-level values (n = 5) rather than 10 independent eye-level observations. The following comparisons were performed: (1) right versus left eyes of mice receiving saline in both eyes; (2) the mean of the two eyes from mice receiving bilateral saline versus saline-treated contralateral eyes of mice receiving unilateral particulate administration; (3) sample #28-treated eyes versus their contralateral saline-treated eyes; (4) sample #30-treated eyes versus their contralateral saline-treated eyes; (5) a post hoc exploratory pooled comparison of all particulate-treated eyes from the sample #28 and #30 groups with their corresponding contralateral saline-treated eyes; and (6) a separate post hoc exploratory between-animal comparison of the pooled particulate-treated eyes with the animal-level mean values from the bilateral-saline control group.

### 2.8 GFAP immunofluorescence staining

To assess retinal glial activation in adult mice, retinal cryosections were immunostained for glial fibrillary acidic protein (GFAP). Sections were incubated with a rabbit anti-GFAP primary antibody (ab7260; Abcam) at a dilution of 1:200, followed by an Alexa Fluor 568-conjugated anti-rabbit secondary antibody (ab175471; Abcam) at a dilution of 1:200. A negative-control slide in which the primary antibody was omitted was included in each staining series. The retina was examined from the posterior pole to the periphery at ×20 magnification. Fluorescence images were acquired using identical settings across the experimental and control groups. GFAP immunoreactivity was assessed qualitatively by an investigator masked to treatment allocation.

### 2.9 Analysis of selected particulate-related elements in ocular tissues

Particulate suspension was instilled into three experimental eyes in each exposed group (#28 and #30), with the contralateral saline-treated eyes used as controls, for eight consecutive days. After euthanasia, the retina–choroid complex was isolated. Manganese and lead were measured by inductively coupled plasma–mass spectrometry through a commercial analytical service (IDEA Co., Ltd., Tokyo, Japan). The analysis was exploratory because only three paired eyes per exposure group were available and only two elements were quantified; therefore, it was not designed to demonstrate particle penetration or tissue accumulation.

### 2.10 In vitro studies of particulate-associated cellular responses

**Fig. 1:**
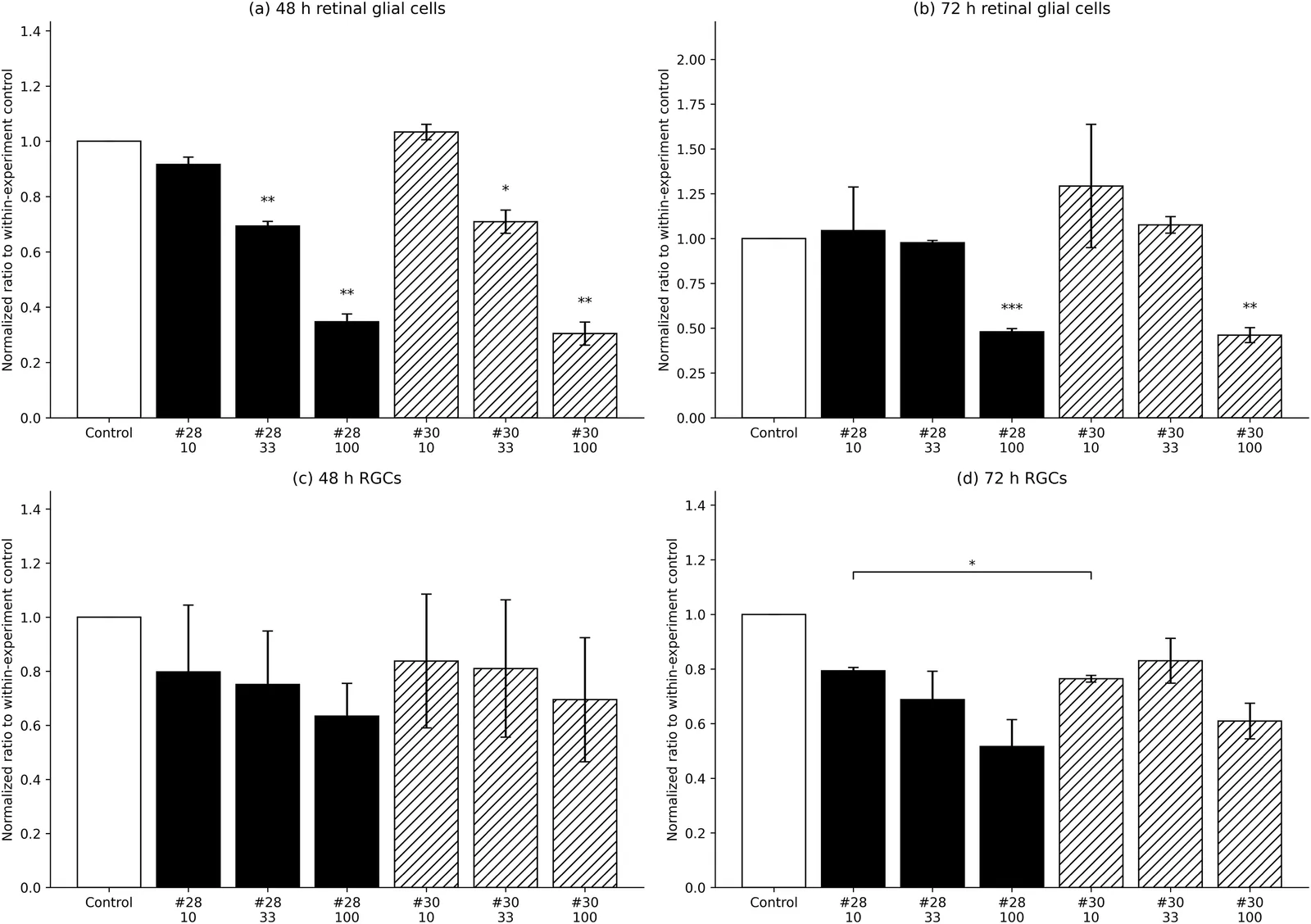
Effects of particulate reference materials on ATP-dependent viability in primary retinal glial cells and retinal ganglion cells. ATP-dependent viability was measured using the CellTiter-Glo assay after exposure to particulate reference materials #28 or #30 at the indicated concentrations. (a) Retinal glial cells after 48 h of exposure. (b) Retinal glial cells after 72 h of exposure. (c) Primary retinal ganglion cells (RGCs) after 48 h of exposure. (d) Primary RGCs after 72 h of exposure. Within each independent biological replicate, technical replicate wells were averaged and each particulate-exposed value was divided by the corresponding vehicle-control mean from the same independent cell isolation, with control defined as 1.0. Bars represent mean ± SE of the normalized biological-replicate values. Biological n: (a) n = 4 for all conditions; (b) n = 2 for 10 and 33 μg/mL and n = 4 for 100 μg/mL; (c) n = 4 for all conditions; (d) n = 2 for 10 and 33 μg/mL and n = 4 for 100 μg/mL. Particulate-exposed conditions were compared with 1.0 using one-sample t-tests with Holm adjustment for six comparisons within each panel. Samples #28 and #30 at the same concentration were compared using paired t-tests with Holm adjustment for three concentration-matched comparisons. Comparisons based on n = 2 were considered exploratory. *P < 0.05, **P < 0.01, and ***P < 0.001 after Holm adjustment.

**Fig. 2:**
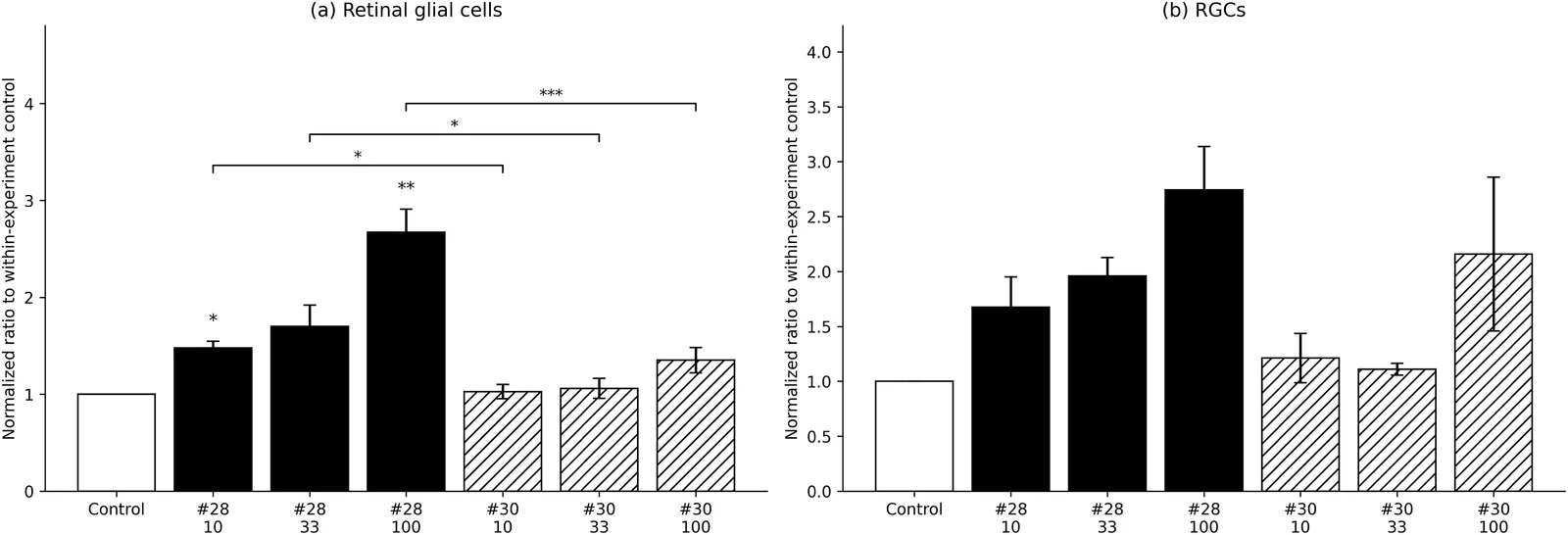
Effects of particulate reference materials on DCFDA fluorescence in primary retinal glial cells and retinal ganglion cells. Intracellular reactive oxygen species-associated fluorescence was assessed using 2′,7′-dichlorofluorescin diacetate (DCFDA) after exposure to particulate reference materials #28 or #30 at the indicated concentrations. (a) Primary retinal glial cells. (b) Primary retinal ganglion cells (RGCs). Within each independent biological replicate, technical replicate wells were averaged and each particulate-exposed value was divided by the corresponding vehicle-control mean from the same independent cell isolation, with control defined as 1.0. Bars represent mean ± SE of normalized biological-replicate values. Biological n: (a) control n = 7; for each sample, n = 5 at 10 μg/mL, n = 4 at 33 μg/mL, and n = 7 at 100 μg/mL; (b) control n = 5; for each sample, n = 5 at 10 μg/mL, n = 2 at 33 μg/mL, and n = 5 at 100 μg/mL. Particulate-exposed conditions were compared with 1.0 using one-sample t-tests with Holm adjustment for six comparisons within each panel. Samples #28 and #30 at the same concentration were compared using paired t-tests with Holm adjustment for three concentration-matched comparisons. Comparisons based on n = 2 were considered exploratory. *P < 0.05, **P < 0.01, and ***P < 0.001 after Holm adjustment.

**Fig. 3:**
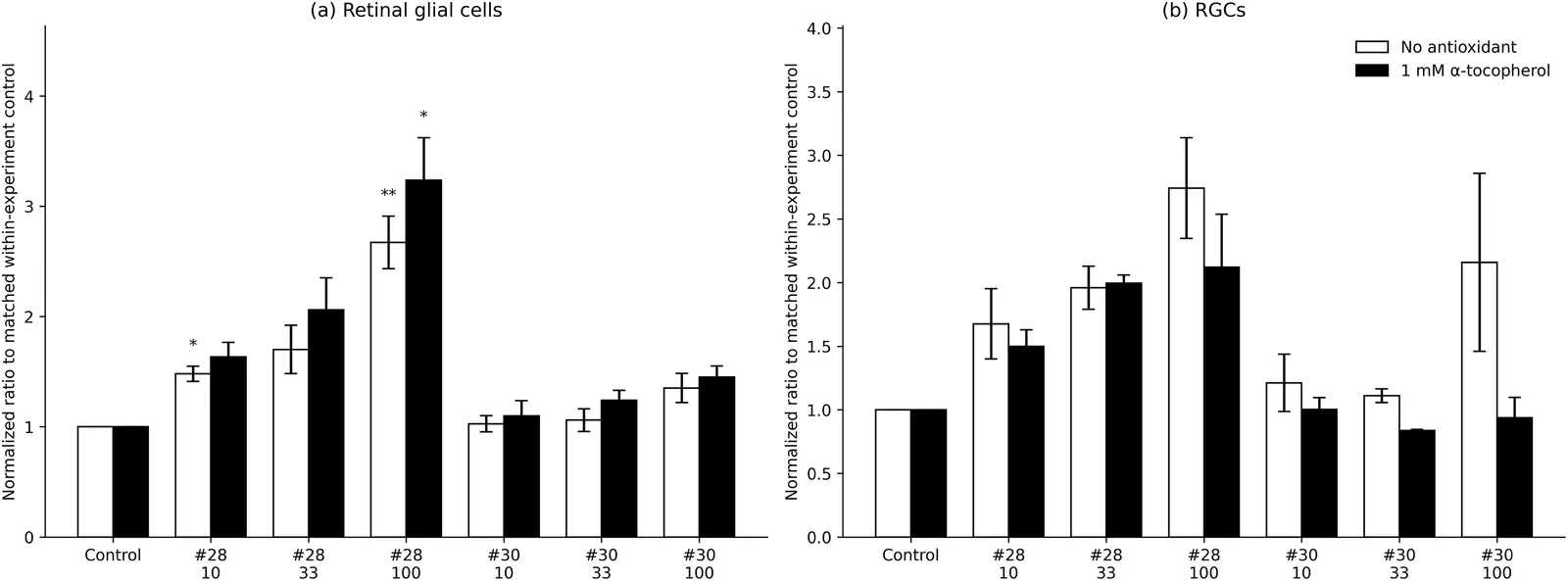
Effects of α-tocopherol on particulate-associated DCFDA fluorescence in primary retinal cells. DCFDA fluorescence was measured after exposure to particulate reference materials #28 or #30 at the indicated concentrations in the absence or presence of 1 mM α-tocopherol. (a) Primary retinal glial cells. (b) Primary retinal ganglion cells (RGCs). Technical replicate wells were averaged within each independent biological replicate before normalization. The no-antioxidant series was normalized to its matched vehicle control, and the α-tocopherol series was independently normalized to its matched α-tocopherol-treated control from the same biological replicate; each matched control was defined as 1.0. Bars represent mean ± SE of normalized biological-replicate values. Biological n for the no-antioxidant series: (a) control n = 7; each sample n = 5 at 10 μg/mL, n = 4 at 33 μg/mL, and n = 7 at 100 μg/mL; (b) control n = 5; each sample n = 5 at 10 μg/mL, n = 2 at 33 μg/mL, and n = 5 at 100 μg/mL. Biological n for the α-tocopherol series: (a) control n = 5; each sample n = 3 at 10 μg/mL, n = 4 at 33 μg/mL, and n = 5 at 100 μg/mL; (b) control n = 3; each sample n = 3 at 10 μg/mL, n = 2 at 33 μg/mL, and n = 3 at 100 μg/mL. The α-tocopherol effect was evaluated by paired t-tests comparing normalized values with and without α-tocopherol at the same particulate sample and concentration, using only matched biological replicates; six P values within each panel were adjusted using the Holm method. Within each treatment series, normalized particulate-exposure values were additionally compared with 1.0 using one-sample t-tests with Holm adjustment. Comparisons based on n = 2 were considered exploratory. *P < 0.05, **P < 0.01, and ***P < 0.001 after Holm adjustment.

**Fig. 4:**
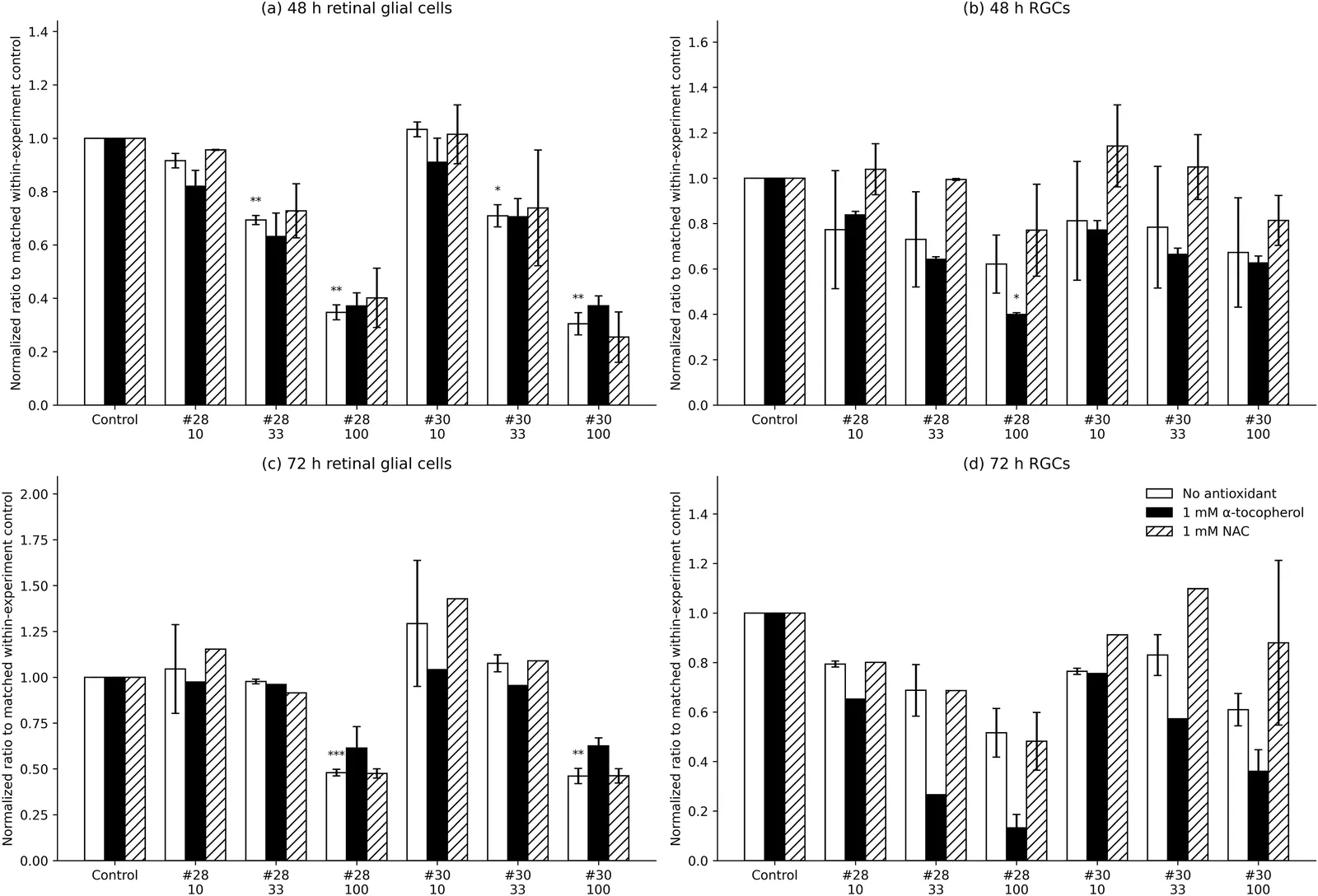
Effects of α-tocopherol and N-acetylcysteine on particulate-associated ATP-dependent viability in primary retinal cells. ATP-dependent viability was measured after exposure to particulate reference materials #28 or #30 at the indicated concentrations in the absence or presence of 1 mM α-tocopherol or 1 mM N-acetylcysteine (NAC). (a) Retinal glial cells after 48 h of exposure. (b) Primary retinal ganglion cells (RGCs) after 48 h of exposure. (c) Retinal glial cells after 72 h of exposure. (d) Primary RGCs after 72 h of exposure. Within each independent biological replicate, technical replicate wells were averaged before normalization. The no-antioxidant, α-tocopherol, and NAC series were each independently normalized to their corresponding within-experiment control, with each matched control defined as 1.0. Bars represent mean ± SE of normalized biological-replicate values. Biological n: (a,b) no antioxidant n = 4 for all conditions, α-tocopherol n = 2 for all conditions, and NAC n = 2 for all conditions; (c,d) no antioxidant n = 4 for control and 100 μg/mL and n = 2 for 10 and 33 μg/mL, whereas α-tocopherol and NAC each had n = 2 for control and 100 μg/mL and n = 1 for 10 and 33 μg/mL. Antioxidant-associated effects were evaluated by paired t-tests comparing normalized particulate-exposure values obtained with α-tocopherol or NAC against the corresponding normalized values obtained without antioxidant treatment at the same particulate sample and concentration. Analyses included only biological replicates in which the relevant matched conditions were available. The six concentration-specific P values were adjusted using the Holm method separately for α-tocopherol and NAC. Within each treatment series, normalized particulate-exposure values were also compared with 1.0 using one-sample t-tests with Holm adjustment. Conditions with n = 1 were displayed descriptively without inferential statistics; comparisons based on n = 2 were considered exploratory. *P < 0.05, **P < 0.01, and ***P < 0.001 after Holm adjustment.

In vitro studies were performed to investigate particulate-associated cellular responses and potential mechanisms. The effects of samples #28 and #30 on retinal glial cells and primary RGCs were evaluated by measuring ATP-dependent cell viability after 48 or 72 h of exposure. For Fig. 1, Fig. 2, Fig. 3, Fig. 4, each block labeled 1st, 2nd, 3rd, and so forth represented an independent cell isolation/experiment and was treated as one biological replicate. Within each biological replicate, the available technical replicate wells for each condition were averaged before statistical analysis. Measurements judged technically unevaluable were excluded without imputation. Consequently, the analyzed biological-replicate n varied by panel and condition; exact n values are reported in the figure legends and supporting data. Technical wells were not treated as independent observations.

### 2.11 RGC isolation

RGCs were isolated using a modified immunopanning–magnetic separation method [[31], [32], [33], [34]]. Briefly, more than 10 eyes were collected from 5-day-old C57BL/6J mice for each independent isolation. After dissociation, the retinal cell suspension was incubated with a rabbit anti-mouse macrophage antibody (CLAD31240; Cedarlane, Burlington, NC, USA) for 5 min and then placed for 30 min in 100-mm Petri dishes coated with goat anti-rabbit IgG (SouthernBiotech, Birmingham, AL, USA), purified mouse anti-CD11b/c antibody (554859; BD Biosciences, Franklin Lakes, NJ, USA), and mouse anti-HNK-1/N-CAM monoclonal antibody (CD57; C6680; Merck, Darmstadt, Germany) to deplete non-RGC populations. The nonadherent retinal cells were then incubated with a biotinylated anti-Thy-1.2 antibody (ab25285; Abcam, Cambridge, UK) for 60 min at 37°C, followed by incubation with anti-biotin MicroBeads for 15 min at 4°C. Magnetically labeled RGCs were collected using a magnetic separation unit (MACS; Miltenyi Biotec, Tokyo, Japan) and cultured in Politi medium. RGC identity was assessed primarily by immunostaining for Brn3a (MAB1585; Merck) and RBPMS (ab194213; Abcam), which were used as RGC markers. Prox1 (925202; BioLegend, San Diego, CA, USA) staining was also examined as an additional retinal-cell marker; because Prox1 is not RGC-specific, it was not used to establish RGC identity. Representative immunofluorescence images of Brn3a-, RBPMS-, and Prox1-stained cultures are provided in Supplemental Fig. 2. Quantitative percentage-positive purity data were not obtained; therefore, RGC culture purity was not quantitatively established.

### 2.12 Retinal glial cell isolation

Primary retinal glial cells were isolated from the retinas of 3-day-old C57BL/6J mice as previously described with minor modifications [[35], [36], [37]]. For each independent isolation, more than 10 eyes were collected under sterile conditions. The retinas were dissected and incubated in 0.2% papain at 37°C for 30 min. The cell suspension was filtered through a 40-μm cell strainer (352340; Falcon, Corning, NY, USA), centrifuged at 1000 rpm for 10 min, and resuspended in DMEM/F12 supplemented with 10% fetal bovine serum (FBS). Cells were seeded onto poly-l-ornithine hydrobromide (P3655; Merck)- and laminin (L2020-1 MG; Merck)-coated dishes and cultured at 37°C in a humidified incubator containing 5% CO_2_. Upon reaching confluence, the cells were passaged. Cells from passages 3–4 were used for subsequent particulate-exposure experiments.

Retinal glial cell identity was verified by immunofluorescence staining using primary antibodies against glutamine synthetase (GS; MAB302, Merck) and glial fibrillary acidic protein (GFAP; ab7260, Abcam). Alexa Fluor® 488 (ab150113; Abcam) and Alexa Fluor® 568 (ab175471; Abcam) were used as secondary antibodies. The cells used for immunofluorescence were maintained in medium supplemented with 5% FBS, human epidermal growth factor (hEGF; E9644-2 MG; Merck, Darmstadt, Germany), and insulin (12585-014; Thermo Fisher Scientific, Waltham, MA). The nuclei were counterstained with DAPI and observed under a fluorescence microscope. Representative glutamine synthetase- and GFAP-stained cultures are shown in Supplemental Fig. 3. Quantitative percentage-positive purity data were not obtained; therefore, retinal glial culture purity was not quantitatively established.

### 2.13 Effects of particulate exposure on ATP-dependent viability in retinal glial cells and primary RGCs

We assessed particulate-associated changes in ATP-dependent viability in retinal glial cells and primary RGCs after 48 or 72 h of exposure. Cells were seeded into white opaque 96-well plates at 1.0 × 10^4^ cells/100 μL/well and exposed to samples #28 or #30 at 10, 33, or 100 μg/mL. Luminescence was measured using the CellTiter-Glo® assay according to the manufacturer's protocol. Because the assay signal depends on both cellular ATP content and the number of viable cells, reductions were interpreted as decreased ATP-dependent viability rather than as specific evidence of mitochondrial dysfunction.

### 2.14 Assessment of reactive oxygen species and antioxidant effects

To assess oxidative stress as a potential contributor to particulate-associated cytotoxicity, intracellular reactive oxygen species (ROS) were evaluated in retinal glial cells and primary RGCs exposed to samples #28 and #30 at 10, 33, or 100 μg/mL. The effects of cotreatment with 1 mM α-tocopherol (T3376-5G; Merck) or 1 mM N-acetylcysteine (NAC; A9165-5G; Merck) for 48 h on ATP-dependent viability were also examined. Antioxidant preparation and concentrations were based on a previous report [38]. α-Tocopherol was dissolved in 100% ethanol at 500 mM, diluted to 100 mM with a mixture of DMEM/F12 containing 20% FBS and serum-free medium (1:1:3), and then diluted 1:100 in culture medium. The final ethanol concentration was approximately 0.2%, and matched vehicle-control wells contained the same ethanol concentration. NAC was dissolved in culture medium at 100 mM and diluted 1:100 before use. Each particulate concentration, including the vehicle control, was evaluated with and without antioxidant treatment. Cell viability was measured by adding 100 μL of CellTiter-Glo® reagent to each well, gently mixing the plates, incubating them at room temperature for 10 min, and measuring luminescence using a SpectraMax ABS Plus microplate reader (Molecular Devices, San Jose, CA, USA). For ROS measurement, retinal glial cells or primary RGCs were seeded at 1.0–1.2 × 10^4^ cells/100 μL/well in black, clear-bottom 96-well plates and incubated with 20 μM 2′,7′-dichlorofluorescin diacetate (DCFDA) from the DCFDA/H2DCFDA Cellular ROS Assay Kit (ab113851; Abcam) at 37°C for 45 min in the dark. After removal of the dye solution, cells were exposed to samples #28 or #30, with or without 1 mM α-tocopherol or 1 mM NAC, for 2 h at 37°C. Fluorescence was measured immediately at excitation/emission wavelengths of 485/535 nm using the SpectraMax ABS Plus. DCFDA fluorescence was normalized to the number of DAPI-positive nuclei in the corresponding wells, and mean fluorescence intensity per cell was calculated using ImageJ.

### 2.15 Effects of cocultured retinal glial cells on RGC survival

As previously reported [34], primary RGCs were cultured on coated round glass coverslips in DMEM/F12 supplemented with 10% FBS for 24 h. Retinal glial cells at passage 3 or 4 were cultured on Millicell membrane inserts (PSHT004R1; Merck) in DMEM/F12 supplemented with 10% FBS for 24 h. After the initial culture, both cell types were rinsed once, and the medium was replaced with Politi medium. The inserts seeded with retinal glial cells were placed above the RGC cultures, followed by incubation in the presence or absence of particulate sample #28 or #30 (100 μg/mL) for 48 h. After coculture, the RGCs were fixed and immunostained with an anti-mouse Brn3a antibody (1:20), followed by an Alexa Fluor 568-conjugated anti-mouse secondary antibody. Nuclei were counterstained with DAPI. Fluorescence images were acquired using a fluorescence microscope, and Brn3a-positive RGCs were quantified using ImageJ.

### 2.16 Statistical analysis

**Fig. 5:**
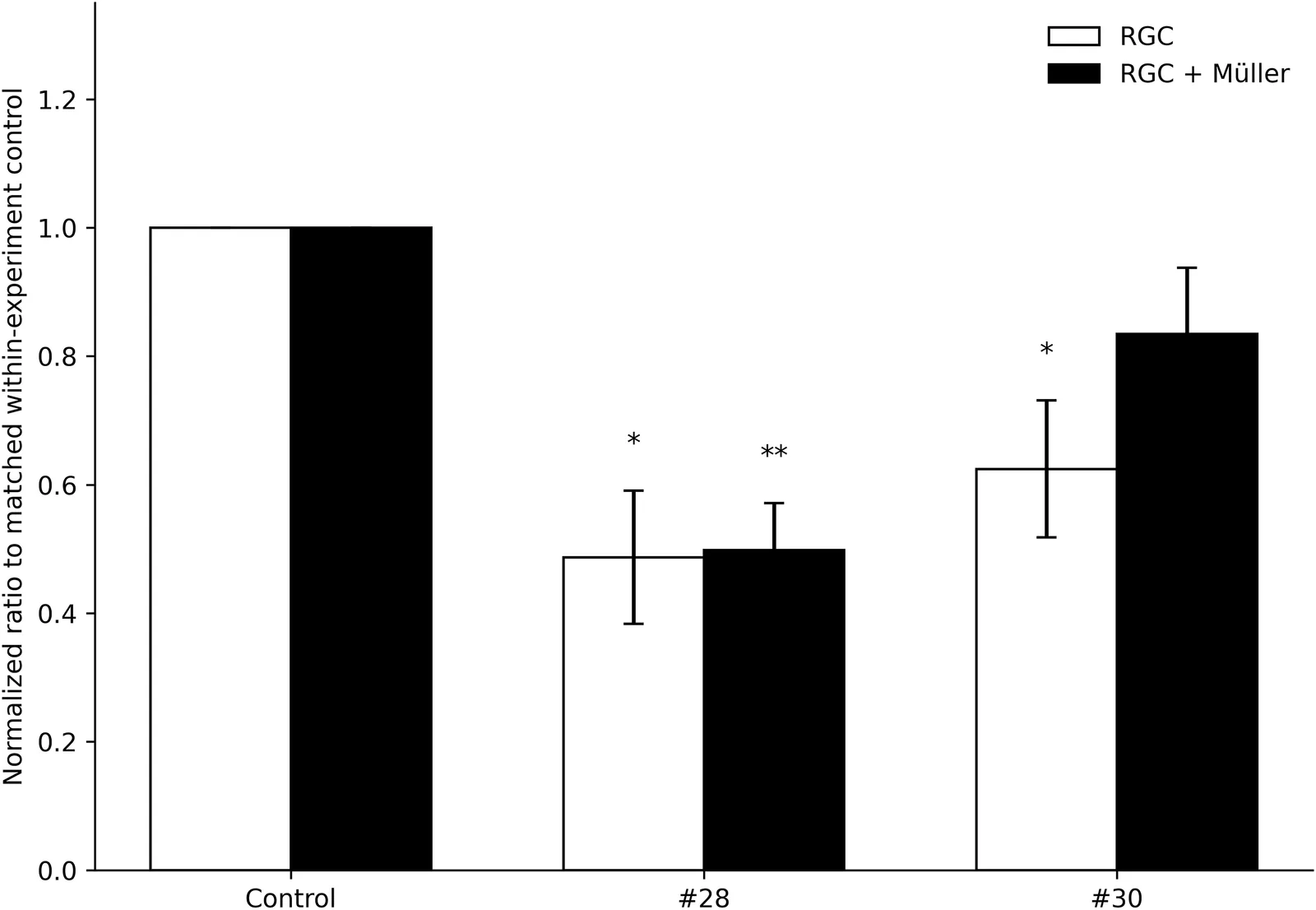
Effects of retinal glial cell coculture on RGC survival following particulate exposure. Primary retinal ganglion cells (RGCs) were cultured alone or cocultured with retinal glial cells and exposed to particulate reference material #28 or #30 at 100 μg/mL for 48 h. For each of five independent experiments, the RGC monoculture series was normalized to the corresponding monoculture control and the coculture series was independently normalized to the corresponding coculture control, with each matched control defined as 1.0. Bars represent mean ± SE of five independent biological replicates. Within each culture condition, normalized values for samples #28 and #30 were compared with 1.0 using one-sample t-tests, with Holm adjustment for the two particulate-exposure comparisons. The coculture effect was assessed by paired t-tests comparing normalized monoculture and coculture values obtained from the same independent experiment for each particulate sample, with Holm adjustment for the two comparisons. Samples #28 and #30 significantly reduced normalized RGC survival in monoculture; in coculture, sample #28 remained significantly below control whereas sample #30 did not. However, direct monoculture-versus-coculture comparisons were not statistically significant for either sample. *P < 0.05 and **P < 0.01 after Holm adjustment.

Statistical analyses other than the reanalysis of Fig. 1, Fig. 2, Fig. 3, Fig. 4, Fig. 5 were performed using SPSS version 31.0 (IBM Corp., Armonk, NY, USA). Fig. 1, Fig. 2, Fig. 3, Fig. 4, Fig. 5 were reanalyzed using Python 3.12 and SciPy 1.17, with the calculations documented in the supporting data. Data are presented as the mean ± standard error (SE) across biological replicates. SE was calculated using the sample standard deviation (n − 1 denominator) divided by the square root of n; SE was not calculated when n = 1. No missing biological-replicate values were imputed. For all in vitro experiments, each independent cell isolation/experiment was considered one biological replicate. Within each biological replicate, measurements from technical replicate wells were first averaged to obtain a single value for each experimental condition. Technical wells were not treated as independent observations.

To minimize between-experiment variation in baseline signal, the biological-replicate means were normalized within each independent experiment before pooling across experiments. For Fig. 1, Fig. 2, the mean value for each particulate-exposed condition was divided by the corresponding vehicle-control mean obtained from the same independent cell isolation, with the within-experiment control defined as 1.0. The resulting normalized ratios, one value per biological replicate and condition, were used for statistical analysis. Each particulate-exposed condition was compared with the theoretical control value of 1.0 using a two-sided one-sample *t*-test. Within each panel, P values from the six particulate-exposure versus control comparisons were adjusted using the Holm method. Samples #28 and #30 were additionally compared at each corresponding concentration using paired t-tests when matched biological replicates were available; the three concentration-matched P values within each panel were adjusted using the Holm method.

For Fig. 3, Fig. 4, each treatment series was normalized to its own corresponding within-experiment control before values from independent experiments were pooled. Values obtained without antioxidant treatment were divided by the matched vehicle-control mean from the same biological replicate, whereas values obtained in the presence of α-tocopherol or N-acetylcysteine (NAC) were divided by their respective antioxidant-treated control mean from the same biological replicate. Thus, each matched control was defined as 1.0. Antioxidant-associated effects were evaluated by paired t-tests comparing normalized particulate-exposure values obtained with antioxidant treatment with the corresponding normalized values obtained without antioxidant treatment, restricted to biological replicates in which both conditions were available. Within each panel, the six concentration-specific P values were adjusted using the Holm method separately for α-tocopherol and NAC. Within each treatment series, normalized particulate-exposure values were also compared with the theoretical value of 1.0 using one-sample t-tests with Holm adjustment.

For Fig. 5, the RGC monoculture series and the RGC–retinal glial cell coculture series were normalized independently to their corresponding within-experiment controls, each defined as 1.0. Samples #28 and #30 within each culture condition were compared with 1.0 using one-sample t-tests, with Holm adjustment for the two particulate-exposure comparisons. The effect of coculture was assessed by paired t-tests comparing the normalized monoculture and coculture values obtained from the same independent experiment for each particulate sample, with Holm adjustment for the two comparisons. The number of biological replicates varied among some experimental conditions because not all concentrations or treatment combinations were evaluated in every independent cell isolation. Statistical comparisons therefore used only biological replicates containing the relevant matched conditions. Comparisons based on very small numbers of biological replicates, particularly n = 1–2, were considered exploratory and were interpreted cautiously. All tests were two-sided, and Holm-adjusted P values < 0.05 were considered statistically significant.

For in vivo analyses, measurements from multiple sections or sampling locations were first aggregated to obtain a single value for each eye. The animal was considered the experimental unit. Comparisons between particulate-treated eyes and contralateral saline-treated eyes were based on within-animal paired differences. A paired *t*-test was used when the paired differences were normally distributed; otherwise, the Wilcoxon signed-rank test was used. To address the possibility that unilateral particulate administration might have affected the contralateral saline-treated eye, a separate post hoc exploratory between-animal comparison was performed using mice treated with saline in both eyes as independent controls. Measurements from the two eyes of each bilateral-saline mouse were averaged to obtain one value per animal. These animal-level mean values were compared with both the saline-treated contralateral eyes and the pooled particulate-treated eyes using Welch's two-sample *t*-test. A post hoc exploratory within-animal analysis was also performed by pooling the sample #28 and #30 groups and comparing the particulate-treated eyes with their corresponding contralateral saline-treated eyes. Elemental analyses were considered exploratory because of the small sample size. All tests were two-sided, and P < 0.05 was considered statistically significant.

## 3 Results

### 3.1 Characterization of primary retinal cell cultures

Representative immunofluorescence images showed Brn3a and RBPMS staining in isolated RGC cultures; Prox1 staining is also shown in Supplemental Fig. 2 but was not interpreted as RGC-specific. Retinal glial cell cultures showed glutamine synthetase and GFAP staining (Supplemental Fig. 3). These images provide qualitative characterization of the cultured cell populations. Because percentage-positive values were not determined, the purity of either culture population was not quantitatively established.

### 3.2 Particulate exposure reduces ATP-dependent viability in primary retinal glial cells and RGCs

After normalization to the corresponding within-experiment control, ATP-dependent viability showed condition-dependent reductions in retinal glial cells (Fig. 1). At 48 h, normalized viability was significantly lower than 1.0 for sample #28 at 33 μg/mL (Holm-adjusted P = 0.0019) and 100 μg/mL (P = 0.0010), and for sample #30 at 33 μg/mL (P = 0.0183) and 100 μg/mL (P = 0.0019). At 72 h, significant reductions were detected at 100 μg/mL for both sample #28 (P = 0.00055) and sample #30 (P = 0.00497). In primary RGCs, none of the particulate-exposure conditions differed significantly from the normalized control after Holm adjustment at either 48 or 72 h. A direct comparison between samples #28 and #30 reached statistical significance only at 10 μg/mL in the 72-h RGC panel (Holm-adjusted P = 0.0287), but this comparison was based on only two matched biological replicates and was considered exploratory.

Thus, the within-experiment control-normalized biological-replicate analysis supported concentration- and time-dependent reductions in ATP-dependent viability, particularly in retinal glial cells, rather than a uniform effect across all tested conditions. The unequal and sometimes small biological-replicate numbers require cautious interpretation.

### 3.3 Particulate exposure increases DCFDA fluorescence

After normalization to the corresponding within-experiment control, DCFDA fluorescence was significantly increased in retinal glial cells exposed to sample #28 at 10 μg/mL (Holm-adjusted P = 0.0108) and 100 μg/mL (P = 0.00248), whereas the other exposure-versus-control comparisons in retinal glial cells and all corresponding comparisons in RGCs were not statistically significant after Holm adjustment (Fig. 2). Direct comparisons between samples #28 and #30 in retinal glial cells were significant at 10 μg/mL (P = 0.0230), 33 μg/mL (P = 0.0230), and 100 μg/mL (P = 0.00020), whereas no sample-specific comparison was significant in RGCs. These findings indicate condition-dependent particulate-associated changes in DCFDA fluorescence but do not by themselves establish oxidative stress as the causal driver of cell injury.

### 3.4 Effects of antioxidants on DCFDA fluorescence and ATP-dependent viability

The effects of α-tocopherol and N-acetylcysteine (NAC) were evaluated after each treatment series had been normalized to its own within-experiment control.

For DCFDA fluorescence, paired comparisons of normalized particulate-exposure values with versus without α-tocopherol did not remain statistically significant after Holm adjustment in either retinal glial cells or RGCs (Fig. 3). In retinal glial cells, several unadjusted comparisons showed numerical reductions with α-tocopherol, but the corresponding Holm-adjusted P values were ≥0.248. In RGCs, no α-tocopherol-associated comparison was significant after adjustment. Within individual treatment series, selected particulate-exposed conditions remained different from their own normalized control value of 1.0, indicating that α-tocopherol did not consistently normalize the particulate-associated response.

For ATP-dependent viability, paired comparisons of normalized particulate-exposure values obtained with α-tocopherol or NAC versus the corresponding values obtained without antioxidant treatment were not statistically significant after Holm adjustment in retinal glial cells or RGCs at either 48 or 72 h (Fig. 4). Several Fig. 4 antioxidant comparisons were based on only one or two matched biological replicates and were therefore considered exploratory. These data do not provide statistically robust evidence that either α-tocopherol or NAC improved particulate-associated ATP-dependent viability under the tested conditions.

### 3.5 Effects of retinal glial cell coculture on RGC survival

After normalization of the monoculture and coculture series to their own corresponding within-experiment controls, normalized RGC survival in monoculture was 0.49 ± 0.10 after sample #28 exposure and 0.62 ± 0.11 after sample #30 exposure (n = 5 each). Both values were significantly below the theoretical control value of 1.0 after Holm adjustment (P = 0.0157 and P = 0.0244, respectively). In coculture, normalized RGC survival was 0.50 ± 0.07 after sample #28 exposure and 0.83 ± 0.10 after sample #30 exposure (n = 5 each); sample #28 remained significantly below 1.0 (Holm-adjusted P = 0.00473), whereas sample #30 did not (P = 0.184). However, direct paired comparisons between normalized monoculture and coculture values were not significant for sample #28 (Holm-adjusted P = 0.920) or sample #30 (P = 0.167) (Fig. 5). Thus, the normalized analysis did not demonstrate a statistically significant coculture-associated improvement in RGC survival for either particulate sample.

### 3.6 Ocular-surface and anterior-segment findings

Apart from the quantified inner retinal changes described below, routine histological examination of postnatal and adult mouse eyes revealed no overt structural abnormalities or inflammatory cell infiltration in the cornea, sclera, iris, ciliary body, lens, or retina following topical administration of either particulate sample. These findings were comparable to those in the control eyes (Supplemental Fig. 1). Because the assessment was based on routine histological examination and did not include molecular or broader immunohistochemical analyses of inflammation, subclinical inflammatory responses cannot be excluded.

### 3.7 Inner retinal thinning and reduced GCL cellularity following topical particulate exposure in postnatal mice

**Fig. 6:**
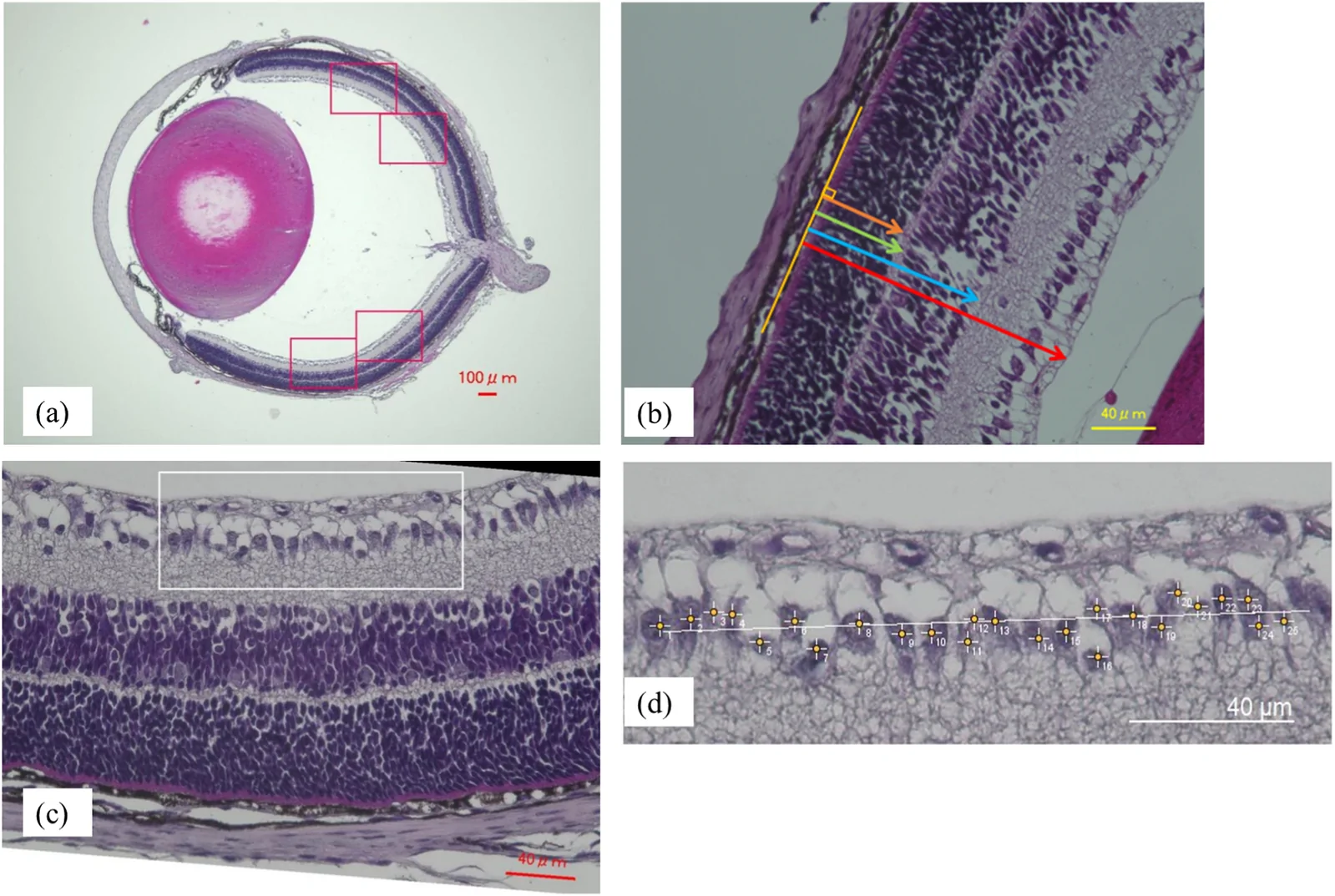
Retinal thickness measurement and GCL cell-counting procedures (a) Representative whole-retinal section showing the four non-overlapping regions selected for analysis (red rectangles). (b) Schematic illustration of retinal-layer thickness measurements. The red, blue, green, and orange arrows indicate the distance from the inner border of the GCL to the outer border of the outer nuclear layer (ONL), the distance from the inner border of the inner nuclear layer to the outer border of the ONL, the distance from the inner border of the outer plexiform layer to the outer border of the ONL, and ONL thickness, respectively. (c) Representative images of the four selected regions used for GCL cell counting; the white rectangle indicates the counting region. (d) Representative manual counting of cells in the GCL. Because H&E staining does not distinguish RGCs from displaced amacrine cells, this analysis represents GCL cellularity rather than an RGC-specific count.

**Fig. 7:**
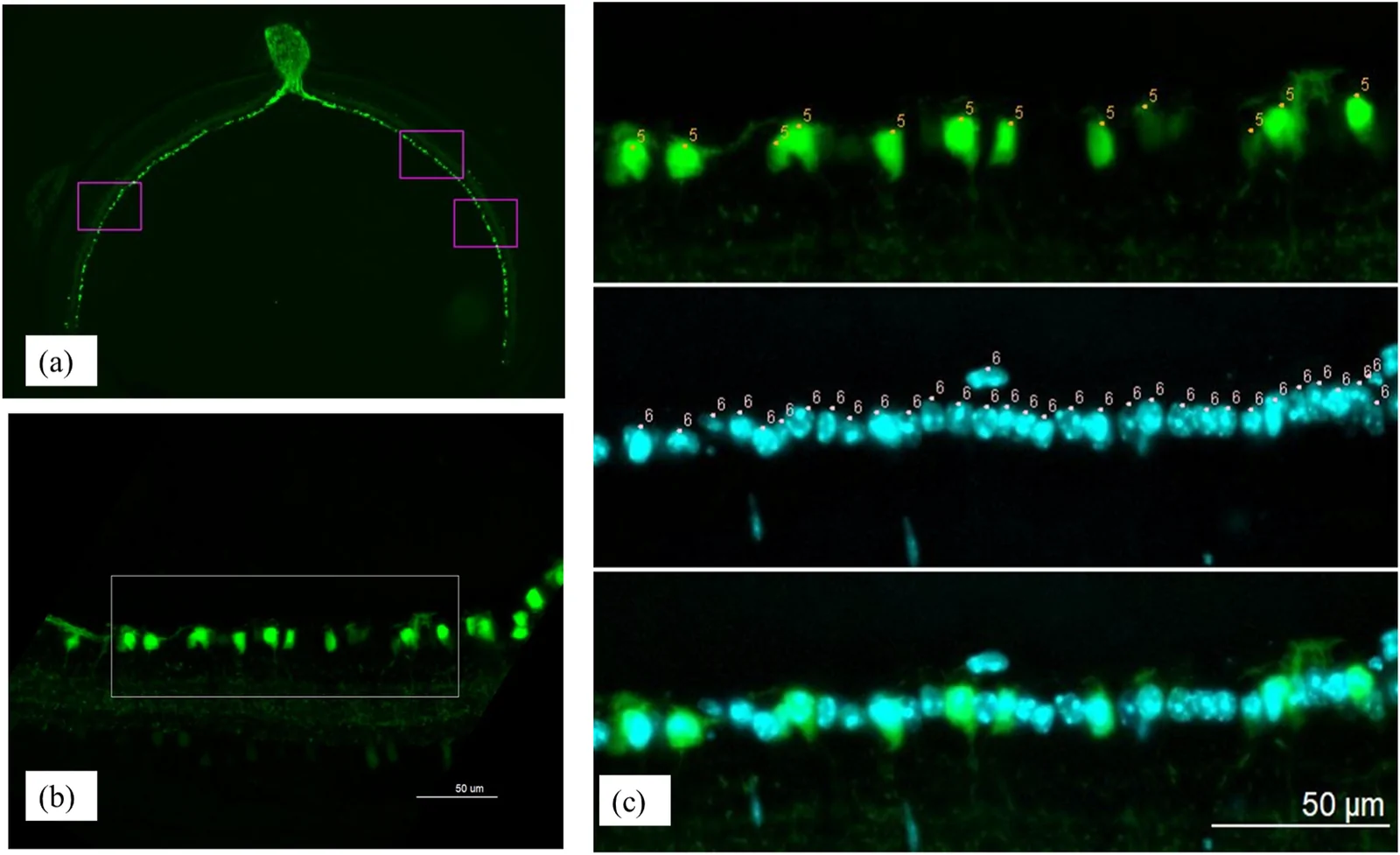
Sampling strategy and RGC quantification in retinal cryosections (a) Fluorescence image of an entire retinal cryosection. Pink rectangles indicate three predefined sampling positions located midway between the optic disc and peripheral retina. (b) Representative images from the three sampling positions; white rectangles (600 pixels wide) indicate the regions used for RGC counting. (c) Representative CFP-positive RGC assessment showing CFP fluorescence (top), DAPI nuclear staining (middle), and the merged image (bottom).

The procedures used for retinal thickness measurement and GCL cell counting and the sampling strategy used for RGC quantification are shown in Fig. 6, Fig. 7, respectively.

**Fig. 8:**
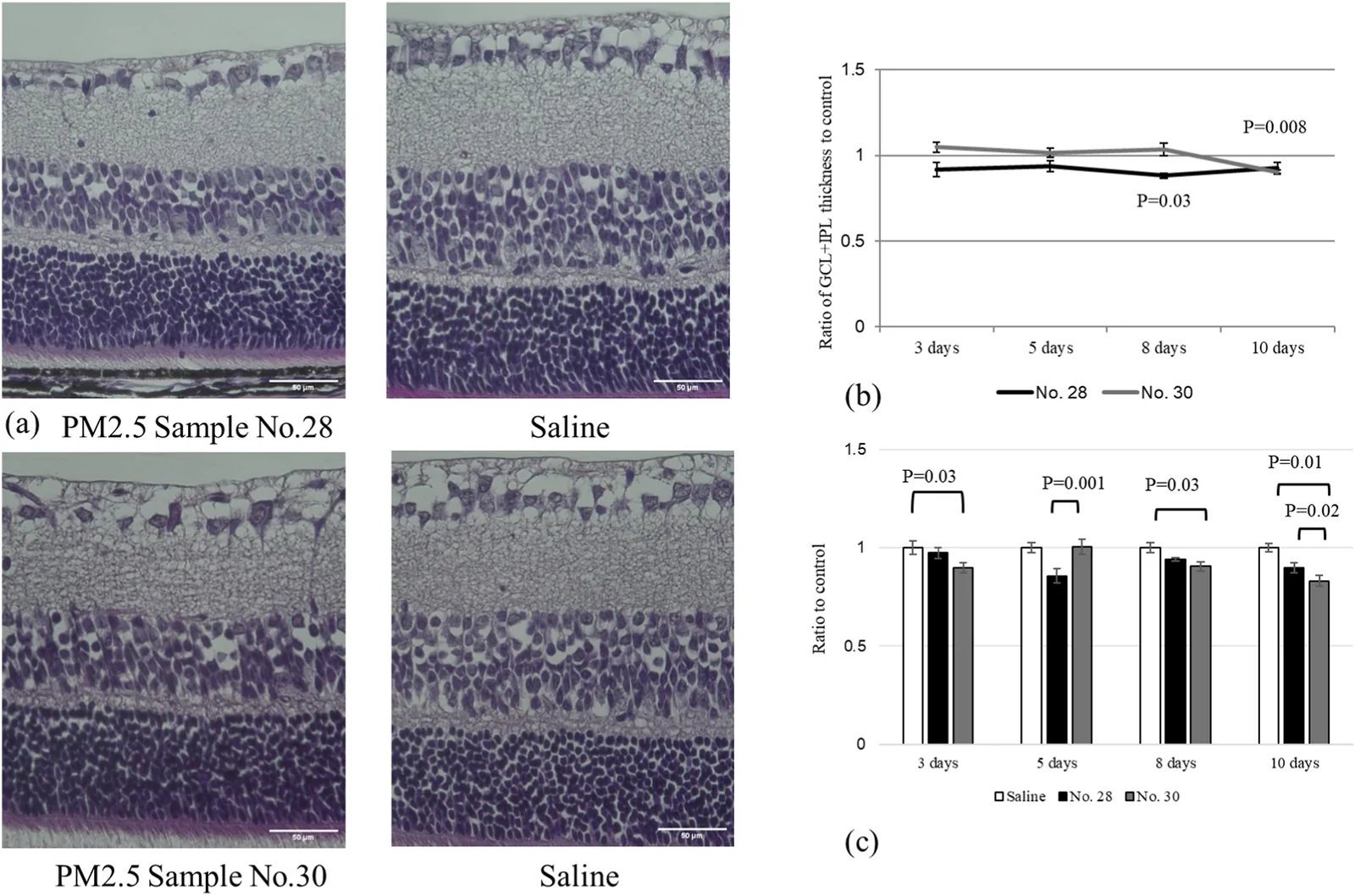
H&E-based assessment of retinal morphology and GCL cellularity following topical particulate administration (a) Representative H&E-stained retinal sections after 10 days of topical particulate or saline administration (scale bar = 50 μm). (b) GCL + IPL thickness relative to that in the contralateral saline-treated eye after 3, 5, 8, and 10 days of exposure. (c) GCL cell counts relative to those in the contralateral saline-treated eye. Because the GCL includes displaced amacrine cells, panel (c) represents GCL cellularity and is not an RGC-specific measurement. The number of animals at each time point is indicated in the figure. Data are presented as the mean ± SE. At each time point, the particulate-treated eye was compared with the contralateral saline-treated eye using the paired analysis specified in the Methods.

Repeated topical instillation of the particulate suspensions was associated with reductions in inner retinal thickness and ganglion cell layer (GCL) cellularity relative to the contralateral saline-treated eye. No gross architectural disruption or inflammatory cell infiltration was detected by H&E staining (Fig. 8a). Quantitative analysis focused on the combined thickness of the GCL and inner plexiform layer (GCL + IPL) and on the number of cells in the GCL.

For sample #28, the GCL + IPL thickness relative to that in the contralateral saline-treated eye was 0.88 ± 0.01 after 8 days of exposure (P = 0.03; n = 5). For sample #30, the corresponding relative value was 0.90 ± 0.01 after 10 days (P = 0.006; n = 3) (Fig. 8b). H&E-based GCL cell counts were also lower in particulate-treated eyes: the relative count was 0.86 ± 0.04 after 5 days of exposure to sample #28 (P = 0.01; n = 7) and 0.90 ± 0.03 after 3 days of exposure to sample #30 (P = 0.03; n = 8) (Fig. 8c). Because the GCL contains displaced amacrine cells in addition to RGCs, these findings represent changes in GCL cellularity and were interpreted together with the RGC-marker analyses.

### 3.8 RGC*-marker findings after prolonged exposure in postnatal mice*

**Fig. 9:**
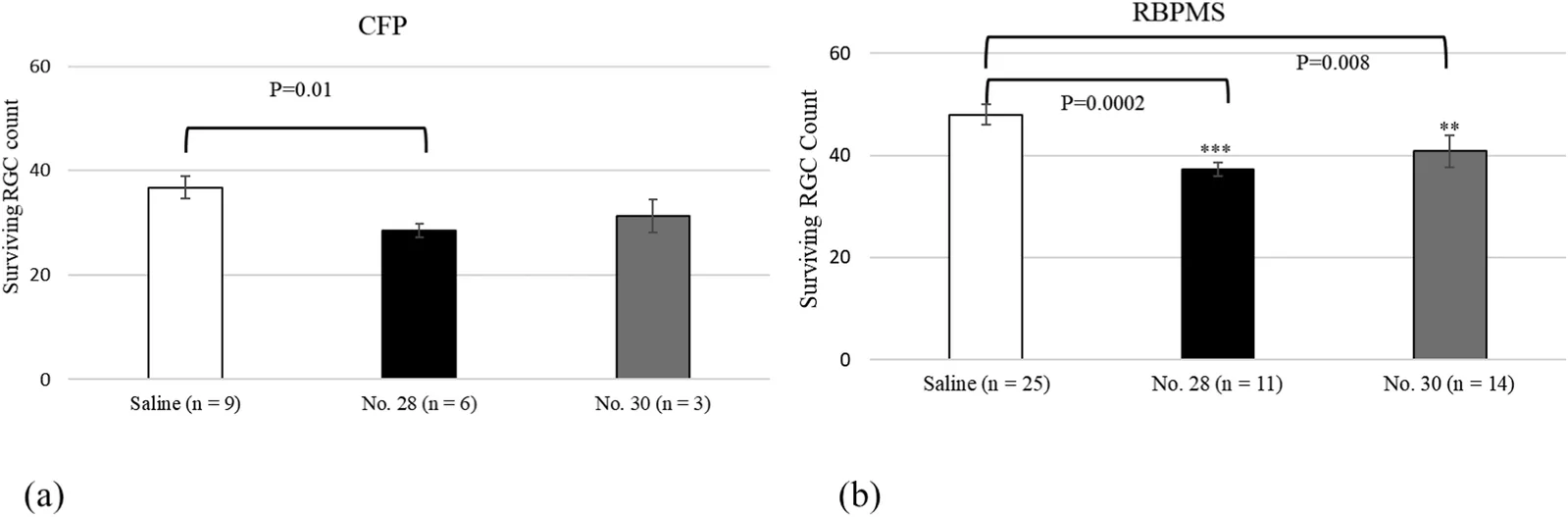
RGC-marker-positive cell counts after prolonged topical particulate administration in postnatal mice (a) CFP-positive cell counts in Thy1-CFP mice and (b) RNA-binding protein with multiple splicing (RBPMS)-positive retinal ganglion cell counts in wild-type mice following 30 days of topical administration of particulate suspension or saline. For the CFP analysis, 12 postnatal Thy1-CFP mice were initially assigned to the sample #28 group (n = 6) or sample #30 group (n = 6). Three mice in the sample #30 group were excluded because tissue-processing problems precluded reliable CFP-positive cell quantification. Consequently, the final CFP analysis included six sample #28-treated eyes, three sample #30-treated eyes, and their nine contralateral saline-treated control eyes. For the RBPMS analysis, the numbers of analyzed eyes were 25 for saline-treated controls, 11 for sample #28, and 14 for sample #30. In both analyses, the saline-treated control eyes were the contralateral eyes of the corresponding particulate-treated eyes, and n denotes the number of animals contributing paired eyes. Data are presented as the mean ± SE. SE values were calculated using the sample standard deviation (n − 1 denominator) divided by the square root of n. Within each particulate group, the particulate-treated eyes were compared with their contralateral saline-treated eyes using the paired statistical analysis described in the Methods.

Of the 12 postnatal Thy1-CFP mice subjected to 30 days of exposure, three mice in the sample #30 group were excluded because tissue-processing problems precluded reliable CFP-positive cell quantification. Consequently, the final CFP analysis included six mice in the sample #28 group and three mice in the sample #30 group, with their nine contralateral saline-treated eyes serving as controls. After 30 days of exposure, CFP-positive cell counts were numerically lower in sample #28-treated eyes (28.5 ± 1.5; n = 6) and sample #30-treated eyes (31.3 ± 3.8; n = 3) than in their contralateral saline-treated eyes (36.8 ± 2.2; n = 9) (Fig. 9a). The small sample size of the sample #30 group limits the precision of this estimate.

RBPMS immunostaining in wild-type mice showed a similar numerical pattern. RBPMS-positive RGC counts were 48.0 ± 1.6 in contralateral saline-treated eyes (n = 25), 37.2 ± 1.9 in sample #28-treated eyes (n = 11), and 40.8 ± 2.0 in sample #30-treated eyes (n = 14) (Fig. 9b).

### 3.9 Retinal effects of topical particulate administration in adult mice

**Fig. 10:**
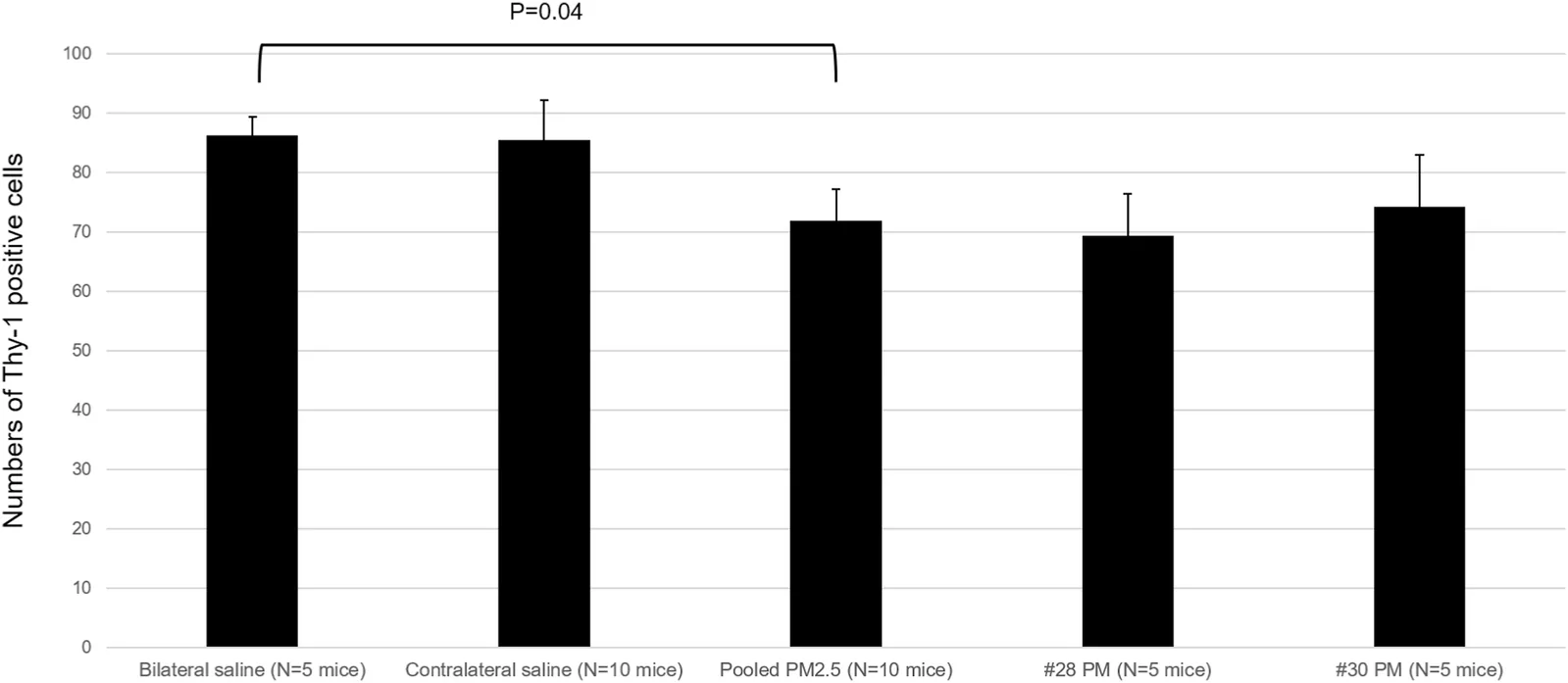
Effects of topical particulate administration on CFP-positive cell counts in adult Thy1-CFP mice Measurements from all analyzed retinal sections were aggregated to yield one value per eye, and each mouse constituted one experimental unit. For the bilateral-saline group, the two eyes of each mouse were averaged, yielding n = 5 animal-level observations rather than n = 10 eye-level observations. No significant difference was observed between the right and left eyes of mice receiving bilateral saline treatment or between the bilateral-saline group and the saline-treated contralateral eyes of mice receiving unilateral particulate administration. For the paired-eye analyses, the paired effect estimate was defined as the within-mouse difference calculated as the CFP-positive cell count in the particulate-treated eye minus that in the contralateral saline-treated eye. The mean paired difference was −8.6 cells for sample #28 (95% CI, −37.5 to 20.3; paired *t*-test, P = 0.455) and −18.6 cells for sample #30 (95% CI, −51.8 to 14.6; paired *t*-test, P = 0.195). In the post hoc exploratory analysis pooling the sample #28 and #30 groups, the mean paired difference was −13.6 cells (95% CI, −30.9 to 3.7; paired *t*-test, P = 0.109). To address the possibility that unilateral particulate administration might have affected the contralateral saline-treated eye, a separate post hoc exploratory between-animal comparison was performed using the bilateral-saline group as an independent control. After averaging the measurements from the two eyes of each bilateral-saline mouse, the mean CFP-positive cell count was 71.8 in the pooled particulate-treated eyes and 86.2 in the bilateral-saline controls. The mean between-group difference, calculated as particulate treatment minus bilateral saline, was −14.4 cells (95% CI, −28.1 to −0.7; Welch's two-sample *t*-test, P = 0.040). Data are presented as the mean ± SE.

There was no significant difference in CFP-positive cell counts between the right and left eyes of mice receiving bilateral saline treatment. CFP-positive cell counts in the bilateral-saline group also did not differ significantly from those in the saline-treated contralateral eyes of mice receiving unilateral particulate administration. In the sample-specific within-mouse analyses, the mean paired difference, calculated as the CFP-positive cell count in the particulate-treated eye minus that in the contralateral saline-treated eye, was −8.6 cells for sample #28 (95% CI, −37.5 to 20.3; paired *t*-test, P = 0.455) and −18.6 cells for sample #30 (95% CI, −51.8 to 14.6; paired *t*-test, P = 0.195). In the post hoc exploratory analysis pooling the sample #28 and #30 groups, the mean paired difference was −13.6 cells (95% CI, −30.9 to 3.7; paired *t*-test, P = 0.109). To address the possibility that unilateral particulate administration might have affected the contralateral saline-treated eye, we performed a separate post hoc exploratory comparison of the pooled particulate-treated eyes with an independent bilateral-saline control group. After averaging the two eyes of each bilateral-saline mouse to obtain one value per animal, the bilateral-saline group comprised n = 5 mice, and the mean CFP-positive cell count was 71.8 in the pooled particulate-treated eyes and 86.2 in the bilateral-saline controls. The mean between-group difference, calculated as particulate treatment minus bilateral saline, was −14.4 cells (95% CI, −28.1 to −0.7; Welch's two-sample *t*-test, P = 0.040) (Fig. 10).

### 3.10 GFAP immunofluorescence staining

**Fig. 11:**
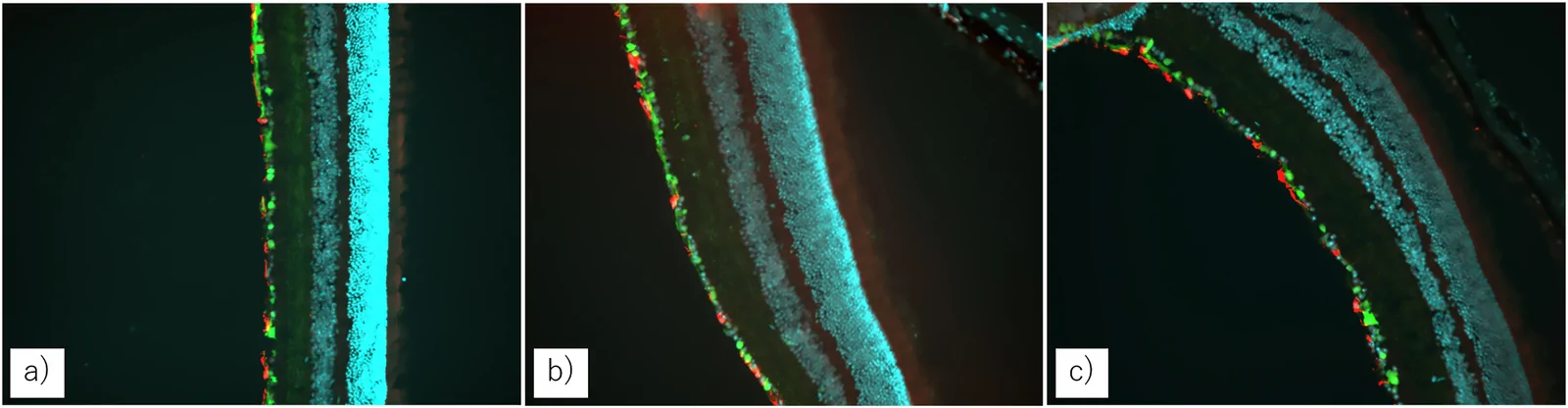
GFAP immunofluorescence staining following topical particulate administration Representative retinal sections from eyes treated with (a) saline, (b) particulate sample #28, and (c) particulate sample #30. GFAP immunoreactivity was confined to the innermost retinal layers and was not observed in deeper retinal layers. Qualitative assessment revealed no apparent differences in staining intensity or distribution among the three groups.

GFAP immunoreactivity was confined to the innermost retinal layers and was not observed in the deeper retinal layers. Qualitative assessment revealed no apparent differences in GFAP staining intensity or distribution among the saline-, sample #28-, and sample #30-treated groups. Representative immunofluorescence images are shown in Fig. 11.

### 3.11 Trace element analysis

Exploratory trace-element analysis did not demonstrate statistically significant differences in retina–choroid manganese or lead concentrations between particulate-exposed and contralateral control eyes (Table 2). The small sample size, detection of these elements in control tissues, and measurement of only two elements preclude conclusions regarding particle penetration, tissue accumulation, or compositional drivers of toxicity.

**Table 2:** Concentrations of selected particulate-related elements in the retina–choroid complex.

Group	Manganese (ng/g)	P value vs control	Lead (ng/g)	P value vs control
Pooled particulate samples	1.04 ± 0.30	0.08	0.15 ± 0.16	0.15
#28	1.09 ± 0.41	>0.2	0.19 ± 0.23	>0.2
#30	0.95 ± 0.20	>0.2	0.12 ± 0.09	>0.2
Contralateral saline	0.75 ± 0.12		0.05 ± 0.01	

Values are presented as the mean ± SE (ng/g). For the individual sample analyses, n = 3 paired eyes per group. The pooled particulate-material analysis combines samples #28 and #30. P values are for paired comparisons with the corresponding contralateral saline-treated eyes.

## 4 Discussion

The principal cellular findings after within-experiment control normalization and biological-replicate analysis were condition-dependent changes in DCFDA fluorescence and ATP-dependent viability. Significant exposure-versus-control differences were concentrated in selected retinal glial-cell conditions, whereas RGC results were less consistent. Importantly, after each antioxidant treatment series was normalized to its own matched control and multiplicity was accounted for, direct comparisons did not show a statistically significant α-tocopherol- or NAC-associated improvement in particulate-exposed conditions. These findings support cellular responses to the particulate reference materials but do not establish oxidative stress as the sole or primary causal mechanism.

The reduction in CellTiter-Glo® signal should also be interpreted cautiously because the assay reflects both cellular ATP and viable cell number. Without normalization to cell count or complementary mitochondrial assays, the data cannot specifically demonstrate mitochondrial dysfunction or ATP depletion on a per-cell basis.

The response patterns differed between retinal cell types. Retinal glial cells showed more consistent significant exposure-versus-control changes in both ATP-dependent viability and DCFDA fluorescence, particularly with sample #28, whereas the RGC findings were generally not significant after multiplicity adjustment. Because the two cell types were not compared in a single prespecified statistical model and several conditions had small biological-replicate numbers, these findings should not be interpreted as definitive evidence of differential intrinsic susceptibility.

The antioxidant experiments require particularly cautious interpretation. Normalizing each antioxidant-treated series to its own matched antioxidant control accounts for experiment-to-experiment baseline variation and separates a particulate-associated effect from any baseline effect of the antioxidant or its vehicle. Under this analysis, the direct α-tocopherol versus no-antioxidant comparisons in Fig. 3 and the α-tocopherol and NAC comparisons in Fig. 4 were not statistically significant after Holm adjustment. Thus, the present data do not provide statistically robust evidence of antioxidant-mediated rescue, although numerical differences in selected conditions may be useful for hypothesis generation.

This revised interpretation is more conservative than analyses based on pooled technical wells or unnormalized absolute signals. The independent cell isolation/experiment, rather than the individual well, was treated as the experimental unit, and technical wells were averaged before within-experiment normalization. This approach avoids pseudoreplication while reducing between-experiment baseline variation. However, normalization does not increase the number of independent biological replicates, and several antioxidant conditions remained underpowered, particularly where only one or two matched experiments were available.

α-Tocopherol is a lipid-soluble radical scavenger within cellular membranes, whereas NAC primarily modulates intracellular glutathione pools and redox signaling [39,40]. The lack of statistically significant antioxidant-associated rescue in the normalized analysis does not exclude a redox contribution to particulate-associated cellular responses; rather, it indicates that the present dataset is insufficient to demonstrate a reproducible protective effect of either intervention. Direct measurements of lipid peroxidation, glutathione status, mitochondrial function, and additional oxidative-stress markers will be required to define mechanism.

Moreover, DCFDA-based ROS measurements can be affected by assay chemistry and redox-active particulate constituents; therefore, changes in fluorescence should be interpreted cautiously. Together, these data emphasize the complexity of particulate-associated redox responses and the need for larger, prospectively balanced biological-replicate experiments before drawing conclusions about antioxidant efficacy.

The coculture analysis was also altered by within-experiment normalization. Although sample #30 exposure was not significantly different from the coculture control, direct paired comparisons of normalized RGC survival between monoculture and coculture were not significant for either sample #28 or #30. Therefore, the present data do not demonstrate a statistically significant protective effect of retinal glial-cell coculture under particulate exposure.

Glial cells are central regulators of retinal homeostasis and contribute to metabolic support, oxidative buffering, and ionic stabilization [34,41]. The absence of a statistically significant coculture effect in the present normalized analysis does not exclude biologically relevant glial–neuronal interactions, but the simplified coculture system and small number of independent experiments limit inference regarding specific protective mechanisms.

Epidemiological studies have associated long-term ambient PM2.5 exposure with glaucoma and retinal neurodegeneration [[15], [16], [17],^20^,^24^,^42^]. Experimental work has additionally shown disruption of the inner blood–retinal barrier and retinal neuronal injury after systemic or airborne exposure [18,20]. The present study extends this literature by evaluating repeated ocular-surface contact with two certified environmental particulate materials and by comparing responses in primary RGCs and retinal glial cells.

Repeated topical administration was associated with inner retinal thinning and lower RGC-marker-positive cell counts in postnatal mice relative to their contralateral saline-treated eyes. In adult Thy1-CFP mice, neither the sample-specific paired comparisons nor the post hoc pooled within-mouse comparison with the contralateral saline-treated eyes was statistically significant. However, in a separate post hoc exploratory between-animal comparison, CFP-positive cell counts were lower in the pooled particulate-treated eyes than in the independent bilateral-saline controls (mean difference, −14.4 cells; 95% CI, −28.1 to −0.7; P = 0.040). This finding should be interpreted cautiously because the comparison was exploratory, the two particulate samples were pooled, and the adult groups were small.

Taken together, these findings support retinal susceptibility following repeated ocular-surface exposure in this experimental model. However, they do not demonstrate that intact particles reached the retina and therefore do not establish the retina as a direct target of deposited particulate matter or define the biological pathway underlying the observed changes.

Ocular-surface deposition is a plausible initial point of contact for airborne particles, but the present experiments did not determine whether intact particles, soluble constituents, inflammatory mediators, or systemic pathways accounted for the retinal changes. The exploratory element analysis did not show significant accumulation and cannot establish transport to the posterior segment. Thus, the biological route linking ocular-surface exposure to retinal findings remains unresolved.

The retinal changes observed in postnatal mice and the more modest findings in adult mice are directionally consistent with epidemiological associations between PM2.5 exposure and glaucoma or retinal neurodegeneration. However, the concentrated instillation paradigm differs substantially from chronic ambient airborne exposure, and the present results should not be interpreted as a quantitative estimate of human risk or as proof of a causal pathway to glaucoma.

This study has several limitations. First, the reference materials were not restricted to particles ≤2.5 μm in diameter; therefore, the experimental results cannot be attributed specifically to a defined PM2.5 fraction. Second, the concentrated instillation paradigm was a proof-of-concept model and did not reproduce the dose, route, frequency, or combined inhalational and ocular exposure encountered in the environment. Third, although an adult-mouse experiment was included, much of the in vivo evidence was obtained during postnatal retinal development, and the adult groups were small; moreover, the pooled adult analysis was conducted post hoc. Fourth, contralateral saline-treated eyes served as within-animal controls, and possible cross-exposure through grooming or systemic redistribution cannot be excluded. Although the independent bilateral-saline group provided an additional control and the exploratory comparison showed fewer CFP-positive cells in the pooled particulate-treated eyes, the small group sizes and post hoc nature of this analysis limit the strength of inference. An independent untreated control group would further strengthen future studies. Fifth, particle penetration, blood–retinal barrier disruption, and the route from the ocular surface to the retina were not directly evaluated. The underpowered trace-element analysis did not demonstrate significant accumulation. Sixth, inflammation was evaluated by routine histology and qualitative GFAP immunofluorescence, but quantitative GFAP analysis, microglial markers, cytokine measurements, and other molecular inflammatory assessments were not performed. Seventh, primary cultures were derived from neonatal mice. Although representative immunostaining images for the selected RGC and glial markers are provided, percentage-positive culture-purity data were not obtained; moreover, the simplified coculture system does not reproduce the cellular architecture of the mature retina. Eighth, CellTiter-Glo® results were not independently normalized to cell number, and functional outcomes such as neurite morphology, electrophysiology, electroretinography, or visual behavior were not assessed. Ninth, technically unevaluable in vitro measurements were excluded without imputation, producing unequal and sometimes very small biological-replicate numbers; several Fig. 4 conditions contained only one biological replicate, for which SE could not be estimated. These conditions are displayed for transparency but provide limited inferential support. Finally, the study was not designed to identify specific toxic constituents.

Future studies should use larger adult cohorts, independent untreated controls, quantitative assessment of glial and inflammatory responses, particle-tracking or blood–retinal barrier analyses, and functional retinal outcomes to determine the mechanism and translational relevance of these observations.

## 5 Conclusion

Direct in vitro exposure of primary mouse retinal cells to two environmental particulate reference materials was associated with condition-dependent changes in DCFDA fluorescence and ATP-dependent viability when each independent experiment was normalized to its own matched control and technical wells were averaged within biological replicates. Significant exposure-associated differences were more consistently observed in retinal glial cells than in RGC cultures. However, after within-experiment normalization and correction for multiple comparisons, direct analyses did not demonstrate a statistically significant protective effect of α-tocopherol or NAC, and retinal glial-cell coculture did not significantly improve RGC survival relative to monoculture. Supporting in vivo experiments showed that repeated ocular-surface exposure was associated with inner retinal thinning and lower RGC-marker-positive cell counts in postnatal mice. In adult mice, the sample-specific and post hoc pooled within-mouse comparisons were not statistically significant, whereas a separate post hoc exploratory between-animal comparison showed fewer CFP-positive cells in pooled particulate-treated eyes than in independent bilateral-saline controls.

These results demonstrate particulate-associated cellular responses and support retinal susceptibility under the experimental conditions used, but the small and unequal biological-replicate numbers limit the strength of the in vitro inference. The findings do not establish particle transport to the retina, a causal oxidative or metabolic mechanism, antioxidant efficacy, or quantitative risk from ambient PM2.5 exposure in humans.

## CRediT authorship contribution statement

**Wanjing Chen:** Writing – original draft, Investigation, Funding acquisition, Formal analysis, Data curation. **Yoko Iizuka:** Writing – review & editing, Methodology, Investigation, Formal analysis. **Fumihiko Mabuchi:** Writing – review & editing, Supervision. **Satoko Yoshizawa:** Writing – review & editing, Methodology, Investigation. **Tomoko Muramatsu:** Writing – review & editing, Investigation. **Kenji Kashiwagi:** Writing – review & editing, Validation, Supervision, Project administration, Methodology, Investigation, Funding acquisition, Formal analysis, Conceptualization.

## Consent to participate

Not applicable. This study did not involve human participants.

## Ethics approval

All animal procedures were conducted in accordance with the ARVO Statement for the Use of Animals in Ophthalmic and Vision Research and were approved by the Institutional Animal Care and Use Committee of the University of Yamanashi (approval No. A3-26). Efforts were made to minimize the number of animals used and animal suffering.

## Consent for publication

Not applicable.

## Declaration of generative AI and AI-assisted technologies in the manuscript preparation process

During the preparation of this work, the authors used ChatGPT (OpenAI) to assist in detecting minor language errors. After using this service, all authors reviewed and edited the content as needed and take full responsibility for the content of the manuscript.

## Funding

This study was partially supported by JST 10.13039/501100025019SPRING (Grant No. JPMJSP2133). The funding source had no role in the study design; collection, analysis, or interpretation of data; writing of the report; or decision to submit the article for publication.

## Declaration of competing interest

The authors declare that they have no competing interests. This study was partially performed with funds provided by JST SPRING, Grant Number JPMJSP2133. The funding source had no role in the study design; in the collection, analysis, or interpretation of data; in the writing of the manuscript; or in the decision to submit the article for publication.

## Data Availability

All numerical data underlying the figures and statistical analyses are provided in the accompanying Supporting Data file. For Fig. 1, Fig. 2, Fig. 3, Fig. 4, Fig. 5, this file includes the raw technical-well measurements, biological-replicate means, within-experiment normalized values, and final statistical results. Numerical data for the in vivo analyses, including the animal-level data used for the adult Thy1-CFP analysis, are also included. Representative microscopy images are provided in the main and supplemental figure files. No data supporting the findings of this study are available only upon request to the authors.

## Appendix A Supplementary data

Fig. S1 Ocular-surface and anterior-segment findings H&E-stained sections of the anterior segment and ocular media. No apparent abnormalities were observed in these structures on routine histological examination. (a) Cornea and lens of an eye treated with sample #28; (b) cornea and lens of an eye treated with sample #30; (c) peripheral cornea, iris, ciliary body, peripheral retina, and lens of an eye treated with sample #28; and (d) the corresponding structures of an eye treated with sample #30.

Fig. S2 Immunofluorescence characterization of primary retinal ganglion cell cultures Representative immunofluorescence images of isolated primary retinal ganglion cell cultures stained for Brn3a (a–c), RNA-binding protein with multiple splicing (RBPMS) (d–f), and Prox1 (g–i). Brn3a and RBPMS were used as RGC identity markers; Prox1 staining is shown as an additional retinal-cell marker and was not interpreted as RGC-specific. Within each triplet, the first panel shows marker immunofluorescence, the second shows DAPI nuclear staining, and the third shows the merged image. These images provide qualitative characterization; percentage-positive culture-purity data were not obtained.

Fig. S3 Immunofluorescence characterization of primary retinal glial cell cultures Representative merged immunofluorescence image of primary retinal glial cell cultures showing (a) glutamine synthetase (GS; red), (b) glial fibrillary acidic protein (GFAP; green), and DAPI (blue). This image provides qualitative characterization; percentage-positive culture-purity data were not obtained.
