# Supplementary material for: Environmental particulate reference materials increase DCFDA fluorescence and reduce ATP-dependent viability in primary mouse retinal cells
Source: Biochem Biophys Rep. 2026 Aug 26;47:102758. doi: 10.1016/j.bbrep.2026.102758 (PMC13544334; doi:10.1016/j.bbrep.2026.102758)
Supplement: Supplementary file 3 [file mmc3.docx]

**Supplemental Figure 3. Immunofluorescence characterization of primary retinal glial cell cultures**

(a) (b)

GFAP + DAPI

GS + DAPI
