# Supplementary material for: Environmental particulate reference materials increase DCFDA fluorescence and reduce ATP-dependent viability in primary mouse retinal cells
Source: Biochem Biophys Rep. 2026 Aug 26;47:102758. doi: 10.1016/j.bbrep.2026.102758 (PMC13544334; doi:10.1016/j.bbrep.2026.102758)
Supplement: Supplementary file 4 [file mmc1.docx]

**Supplemental** **Figure 1 Ocular surface and anterior segment findings**

(a)

(b)

(c)

(d)
